# Alcohol consumption and epigenetic age acceleration across human adulthood

**DOI:** 10.18632/aging.205153

**Published:** 2023-10-26

**Authors:** Mengyao Wang, Yi Li, Meng Lai, Drew R. Nannini, Lifang Hou, Roby Joehanes, Tianxiao Huan, Daniel Levy, Jiantao Ma, Chunyu Liu

**Affiliations:** 1 Department of Biostatistics, Boston University School of Public Health, Boston, MA 02118, USA; 2 Department of Preventive Medicine, Northwestern University Feinberg School of Medicine, Chicago, IL 60611, USA; 3 Population Sciences Branch, Division of Intramural Research, National Heart, Lung, and Blood Institute, National Institutes of Health, Bethesda, MD 20892, USA; 4 Framingham Heart Study, Framingham, MA 01702, USA; 5 Nutrition Epidemiology and Data Science, Friedman School of Nutrition Science and Policy, Tufts University, Boston, MA 02111, USA

**Keywords:** alcohol consumption, epigenetic aging, DNA methylation, hypertension

## Abstract

The alcohol-associated biological aging remains to be studied across adulthood. We conducted linear regression analyses to investigate the associations between alcohol consumption and two DNA methylation-based biological age acceleration metrics in 3823 Framingham Heart Study participants (24–92 years and 53.8% women) adjusting for covariates. We also investigated whether the two epigenetic aging metrics mediated the association of alcohol consumption with hypertension. We found that higher long-term average alcohol consumption was significantly associated with biological age acceleration assessed by GrimAge acceleration (GAA) and PhenoAge acceleration (PAA) in middle-aged (45–64 years, *n* = 1866) and older (65–92 years, *n* = 1267) participants while not in young participants (24–44 years, *n* = 690). For example, one additional standard drink of alcohol (~14 grams of ethanol per day) was associated with a 0.71 ± 0.15-year (*p* = 2.1e-6) and 0.60 ± 0.18-year (*p* = 7.5e-4) increase in PAA in middle-aged and older participants, respectively, but the association was not significant in young participants (*p* = 0.23). One additional standard serving of liquor (~14 grams of ethanol) was associated with a greater increase in GAA (0.82-year, *p* = 4.8e-4) and PAA (1.45-year, *p* = 7.4e-5) than beer (GAA: 0.45-year, *p* = 5.2e-4; PAA: 0.48-year, *p* = 0.02) and wine (GAA: 0.51-year, *p* = 0.02; PAA: 0.91-year, *p* = 0.008) in middle-aged participant group. We observed that up to 28% of the association between alcohol consumption and hypertension was mediated by GAA or PAA in the pooled sample. Our findings suggest that alcohol consumption is associated with greater biological aging quantified by epigenetic aging metrics, which may mediate the association of alcohol consumption with quantitative traits, such as hypertension.

## INTRODUCTION

The effect of alcohol consumption on health, including cardiovascular disease (CVD) and cancer, is complex and has been discussed previously [1, 2]. According to the Centers for Disease Control and Prevention (CDC), excessive alcohol use, including binge drinking, heavy drinking, underage drinking, and any alcohol use during pregnancy, causes over 140,000 deaths in the US annually [3, 4]. Light and moderate drinking are previously thought to have protective benefits for CVD and type 2 diabetes [5, 6]. However, recent studies with meta-analyses suggest there is no safe level of alcohol on human health [7, 8].

DNA methylation (DNAm) refers to the addition of a methyl group to the carbon 5 position of cytosine at cytosine-phosphate-guanine (CpG) sites, where a cytosine nucleotide and a guanine nucleotide in the single strand are linked by a phosphate group [9]. DNAm levels are measured with the bisulfite conversion method after DNA extraction and purification from whole blood samples [10]. DNAm age, or epigenetic age, is a calculated biological age based on DNAm measurements. Previous studies have shown epigenetic age is a robust biomarker of chronological age [11–15]. Multiple generations of epigenetic age variables have been constructed using DNAm to predict aging-related diseases and mortality from different perspectives. The first-generation epigenetic ages (Horvath’s age, Hannum’s age, and skin and blood clock) select CpGs based on chronological age, while the second-generation epigenetic ages (GrimAge and PhenoAge) incorporate clinical biomarkers [11–15]. Each DNAm age has its corresponding epigenetic age acceleration (EAA) to indicate the discrepancy between chronological age and DNAm age. A positive value of EAA indicates a more advanced biological age relative to the chronological age and vice versa.

Previous studies have examined the association between alcohol and epigenetic aging metrics [16–20]. A recent study identified cumulative alcohol consumption and recent binge drinking were associated with GrimAge acceleration (GAA) in young adults (32–49 years) [16]. Similar findings were also observed among older African Americans [17] and non-Hispanic White women [18]. Moreover, two previous studies demonstrated that the alcohol use disorder was associated with Horvath’s age acceleration and PhenoAge acceleration (PAA) [19, 20]. Hypertension, which was often linked to chronic alcohol consumption, was also found to be positively associated with Horvath’s age acceleration in the elderly [21, 22]. The hypertensive target organ damage (e.g., higher left ventricular mass index) was also associated with Intrinsic epigenetic age acceleration (IEAA) and extrinsic epigenetic age acceleration (EEAA) in older African Americans [23]. Motivated by these previous studies, we aimed to explore the association of long-term average and cross-sectional alcohol consumption with EAAs in 3823 participants of the Framingham Heart Study (FHS). We investigated associations of total alcohol consumption and different types of alcoholic beverage consumption with EAAs throughout adulthood (24–94 years). We also conducted a mediation analysis to explore whether EAAs mediated the association of long-term average alcohol consumption with hypertension, an important risk factor for CVD.

## RESULTS

### Sample characteristics

This study included 3823 participants with both DNAm measurements and alcohol consumption (Table 1). The three age groups contained similar proportions of women and men. Older participants (> 64 years) tended to have a lower education level (*p* < 0.001) compared to young (22–44 years) and middle-aged (45–64 years) groups (Table 1). For example, about 31.8% of participants in the older group, 47.5% of participants in the middle-aged group, and 63.5% of participants in the young group were 4-year college graduates. Additionally, the older group (80.5%) had a higher prevalence of hypertension than that in the young (29.1%) and middle-aged (56.1%) groups (*p* < 0.001) (Table 1).

**Table 1:** Participant characteristics in age groups in the Framingham Heart Study.

**Variable** **Mean (SD) or *n* (%)**	**Young**	**Middle-aged**	**Older**	***P* value**
	**24–44 years**	**45–64 years**	**65–92 years**	
	**(*n* = 690)**	**(*n* = 1866)**	**(*n* = 1267)**	
Generation				<0.001
Offspring	12 (1.7%)	1051 (56.2%)	1258 (99.3%)	
Third Generation	678 (98.3%)	815 (43.8%)	9 (0.7%)	
Women	377 (54.6%)	990 (53.1%)	689 (54.4%)	0.68
Age, years	38.5 (4.7)	55.3 (5.8)	73.0 (5.8)	<0.001
BMI, kg/m^2^	27.1 (5.6)	28.5 (5.8)	28.0 (5.0)	<0.001
Smoke pack-year	1.30 (4.9)	2.94 (10.6)	1.67 (9.9)	<0.001
Physical activity	39.3 (15.9)	38.8 (15.2)	36.9 (13.5)	<0.001
Education				<0.001
Under college	62 (9.0%)	395 (21.2%)	479 (37.8%)	
Some college	190 (27.5%)	584 (31.3%)	385 (30.4%)	
College and above	438 (63.5%)	887 (47.5%)	403 (31.8%)	
Hypertension	201 (29.1%)	1046 (56.1%)	1020 (80.5%)	<0.001
Alcohol consumption variables				
Long-term consumption^1^				
Continuous, drinks/day				
Beer	0.07 (0, 0.23)	0.07 (0, 0.29)	0.04 (0, 0.20)	0.01
Wine	0.07 (0, 0.21)	0.09 (0.02, 0.30)	0.13 (0.03, 0.38)	<0.001
Liquor	0.02 (0, 0.07)	0.04 (0, 0.13)	0.08 (0.02, 0.27)	<0.001
Total	0.29 (0.11, 0.55)	0.39 (0.12, 0.89)	0.46 (0.14, 1.15)	<0.001
Categorical				<0.001
Non-drinker	74 (10.7%)	172 (9.2%)	71 (5.6%)	
Light drinker	595 (86.2%)	1463 (78.4%)	975 (77.0%)	
At-risk drinker	16 (2.3%)	166 (8.9%)	150 (11.8%)	
Heavy drinker	5 (0.7%)	65 (3.5%)	71 (5.6%)	

Median with quantiles was provided for continuous alcohol consumption variables. ^1^Long-term average drinking was calculated as the average of consumption (total alcohol or each type of alcoholic beverages) up to 26 years. Four drinking categories were defined with the continuous long-term average alcohol consumption. Non-drinkers were those without any alcohol consumption. Light drinkers were those who consumed alcohol less than 1 drink per day for women and less than 2 drinks per day for men; at risk drinkers were those who consumed alcohol 1–2 drinks per day for women and 2–3 drinks per day for men; heavy drinkers were those who consumed alcohol more than 2 drinks per day for women and more than 3 drinks per day for men.

A higher proportion of heavy drinkers (> 2 drinks per day for women and > 3 drinks per day for men) was found in the older group (5.6%) compared to middle-aged (3.5%) and younger (0.7%) groups based on the average total alcohol consumption over long-term (*p* < 0.001). Greater long-term average consumption of wine and liquor was observed in middle-aged and older participants compared to young participants, while the same trend was not observed with long-term average beer consumption.

### Association of long-term average alcohol consumption with GAA and PAA

**Figure 1:**
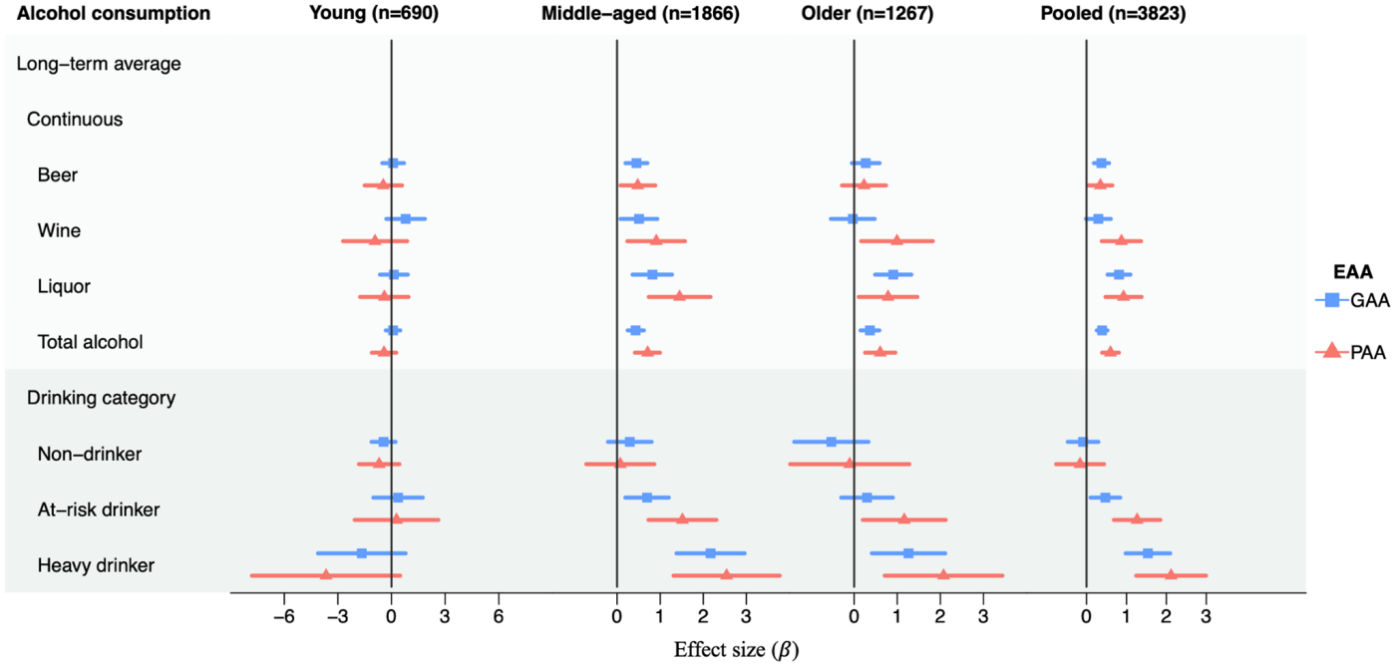
**Association analyses between long-term average alcohol consumption and EAAs in each age group and in pooled samples in the Framingham Heart Study.** Age groups: young (24–44 years), middle-aged (45–64 years), older (65–94 years). The x-axis represents the effect size of alcohol consumption on GAA or PAA. Results are adjusted for sex, physical activity score, education level, BMI, smoke pack-year, chronological age, and lab. The long-term average drinking was calculated as the average of consumption (total alcohol or each type of alcoholic beverages) across up to 26 years. For continuous consumption variables, the effect size was in response to a standard drink per day. The drinking category was grouped based on the long-term average alcohol consumption, with light drinkers as the reference. Non-drinkers were defined as participants with average alcohol consumption equal to zero; light drinkers were defined as less than 1 drink per day for women and less than 2 drinks per day for men; at risk drinkers were defined as 1–2 drinks per day for women and 2–3 drinks per day for men; heavy drinkers were defined as more than 2 drinks per day for women and more than 3 drinks per day for men. Abbreviations: GAA: GrimAge acceleration; PAA: PhenoAge acceleration. Effect sizes and *p*-values can be found in Supplementary Tables 2, 3.

Before conducting association analyses in each age group, we first investigated the interaction between EAA and three age groups. A significant interaction was observed between long-term average alcohol consumption and age groups for PAA (*p* = 9.4e-4) but not for GAA (Supplementary Table 1). To be consistent, we explored the associations of alcohol consumption with PAA and GAA within the age groups. In the primary analyses, we quantified an association of long-term average alcohol consumption (the main predictor variable) with each EAA metric (the outcome variable) in linear regression, adjusting for sex, physical activity index (PAI), education level, body mass index (BMI), smoking (pack-year), chronological age, and lab index (for batch effects) as covariates. We observed significant associations of the long-term average alcohol consumption with GAA and PAA in middle-aged and older age groups, while not in the young age group (Figure 1). For example, one additional drink of total alcohol consumption per day was associated with a 0.43-year increase in GAA in middle-aged participants (*p* = 5.4e-6) and a 0.37-year increase in GAA in older participants (*p* = 0.001). Similarly, one additional drink of total alcohol consumption per day was associated with a 0.71-year increase in PAA in middle-aged participants (*p* = 2.1e-6) and a 0.60-year increase in PAA in older participants (*p* = 7.5e-4). No significant association was observed between long-term average total alcohol consumption and the other three EAA metrics (IEAA, EEAA, and EAASkinBlood) in any of the three age groups (Supplementary Table 2).

To investigate if EAA displayed a linear relationship with alcohol consumption in the age groups, we compared EAA metrics in non-drinkers, at-risk drinkers, and heavy drinkers with light drinkers defined using long-term average total alcohol consumption (Figure 1). Categorical alcohol consumption variables were defined based on the following rules: non-drinkers included participants with average total alcohol consumption equal to zero; light drinkers included women consuming < 1 drink per day and men consuming < 2 drinks per day; at-risk drinkers included women consuming 1–2 drinks per day and men consuming 2–3 drinks per day; heavy drinkers contained women consuming > 2 drinks per day and men consuming > 3 drinks per day. We observed that heavy drinkers displayed a stronger association of GAA and PAA than at-risk drinkers in middle-aged and older participants, while non-drinkers possessed a similar GAA and PAA as light drinkers. As shown in Figure 1, the relationship between total alcohol consumption and age acceleration is linear for both GAA and PAA. For example, compared to light drinkers, we observed an increase in GAA in at-risk drinkers (β = 0.70, *p* = 0.007) and in heavy drinkers (β = 2.17, *p* = 8.0e-8) in the middle-age group. Similarly, compared to light drinkers, we observed a 1.52-year increase in PAA in at-risk drinkers (*p* = 1.6e-4) and a 2.54-year increase in PAA in heavy drinkers (*p* = 5.4e-5) in the middle-age group (Supplementary Table 3).

### Association of epigenetic age acceleration with different alcoholic beverages

For each type of alcoholic beverage (i.e., beer, wine, and liquor), we explored its association with EAAs using the same statistical models with the same set of covariates adjusted for the analyses of long-term average total alcohol consumption. We observed that three types of alcoholic beverages displayed different effect sizes in association analyses with GAA and PAA in middle-aged and older participants (Figure 1). Both wine (β = 0.91, *p* = 0.008) and liquor (β = 1.45, *p* = 7.4e-5) consumptions were associated with PAA, while no significant association was observed between beer consumption and PAA in middle-aged participants (Supplementary Table 2). In addition, liquor consumption displayed the greatest accelerative change compared to the other two alcoholic beverages (Figure 1). For example, we observed a 0.82-year increase in GAA with one additional drink of liquor consumption per day in middle-aged participants (*p* = 4.8e-4) and a smaller increase in GAA with one additional drink of beer consumption per day (β = 0.45, *p* = 5.2e-4) (Supplementary Table 2). The similar trend could also be observed in older participants. Compared to a 0.91-year increase in GAA with one additional drink of liquor consumption per day (*p* = 2.5e-5) in older participants, smaller increases were observed in GAA with one addition drink of beer or wine (beer: β = 0.27, *p* = 0.10; wine: β = −0.03, *p* = 0.90) (Supplementary Table 2).

### Secondary analyses of alcohol consumption variables with epigenetic age acceleration

#### Association of cross-sectional alcohol consumption with epigenetic age acceleration

Instead of long-term average alcohol consumption, we analyzed the association utilizing cross-sectional alcohol consumption data derived from the same exam when DNA samples were collected. The cross-sectional alcohol consumption varied in three age groups. The highest cross-sectional beer and wine consumption was observed in the young group, while the cross-sectional liquor consumption was close to zero in all three age groups, and a similar cross-sectional total alcohol consumption was observed in both young and middle-aged groups (Supplementary Table 4).

Cross-sectional alcohol consumption variables (i.e., total alcohol and each type of alcoholic beverages) demonstrated similar associations with both GAA and PAA, while the effect sizes became much smaller compared to long-term average alcohol consumption variables (Supplementary Figure 1). For example, in middle-aged participants, one additional drink of total alcohol consumption per day was associated with 0.71 (long-term average, *p* = 2.1e-6) and 0.47 (cross-sectional, *p* = 4.0e-6) year increase in PAA; one additional drink of liquor consumption per day was associated with 1.45 (long-term average) and 0.59 (cross-sectional) year increase with PAA (Supplementary Tables 2, 5). Of note, cross-sectional and long-term average alcohol consumption displayed different associations with GAA. Both long-term average liquor consumption (β = 0.91, *p* = 2.5e-5) and total alcohol consumption (β = 0.37, *p* = 0.001) were significantly associated with increases in GAA in the older participants, while cross-sectional liquor and total alcohol consumption were not significantly associated with GAA. For the other three EAA metrics (IEAA, EEAA, and EAASkinBlood), the only significant association observed was between cross-sectional wine consumption and IEAA (β = −1.11, *p* = 0.004) in the young participants (Supplementary Table 5).

#### Association of recent binge drinking with epigenetic age acceleration

We identified 1167 of 3823 participants with recent binge drinking behavior, which was defined as women consuming over 4 drinks per day, men consuming over 5 drinks per day, or those with less than 2 alcohol-free days per week. Young participants contained the greatest proportion of people with recent binge drinking (39.7%), compared to middle-aged (31.2%) and older participants (24.5%) (*p* < 0.001) (Supplementary Table 4). We also found that 87.2% of long-term average heavy drinkers and 76.2% of long-term average at-risk drinkers had recent binge drinking. In contrast, only 0.6% of non-drinkers and 26.0% of light drinkers had recent binge drinking (Supplementary Table 6).

Recent binge drinking displayed significant associations with GAA and PAA, similar to what we observed with cross-sectional total alcohol consumption (Supplementary Figure 1). For example, middle-aged participants with recent binge drinking displayed a 0.56-year increase in GAA (*p* = 4.5e-4) and a 0.93-year increase in PAA (*p* = 1.8e-4) compared to those without recent binge drinking (Supplementary Table 7).

#### Alcohol consumption on epigenetic age acceleration in pooled samples

We conducted association analyses of long-term average and cross-sectional alcohol consumption with EAA metrics in pooled participants of all age groups. We observed mostly consistent associations of long-term average and cross-sectional alcohol consumption variables with PAA and GAA in pooled samples compared to the results found in middle-aged and older age groups (Figure 1, Supplementary Figure 1). Using associations with long-term average total alcohol consumption as an example, one additional drink of total alcohol consumption per day was associated with a 0.43-year increase in GAA in the middle-aged group and a 0.37-year increase in GAA in the older age group. Similarly, one additional drink of long-term average total alcohol consumption per day was associated with a 0.39-year increase in GAA (*p* = 1.3e-8) in the pooled sample (Supplementary Table 2). Although we observed consistent associations of alcohol consumption variables with GAA and PAA in the pooled sample compared to middle-aged and older groups, differences still existed in association analyses with the pooled sample. For example, one additional drink of cross-sectional liquor consumption per day displayed a 0.31-year increase in GAA in the pooled sample (*p* = 0.002) while such association was not observed in any age group (Supplementary Table 5).

#### Association of long-term average alcohol consumption with epigenetic age acceleration adjusting for white blood cell compositions

To investigate whether white blood cell (WBC) compositions may confound the associations between long-term alcohol consumption and EAAs, we further adjusted for imputed cell compositions, including CD8+ T cells, CD4+ T cells, natural killer cells, B cells, monocytes, and granulocytes. Compared to results in primary analyses, we observed mostly consistent results from the association analysis with WBC composition adjustment (Pearson correlation r > 0.9) (Supplementary Figure 2). Of note, four associations were not significant (*p* < 0.01) before adjusting for WBC compositions but became significant with effect sizes increasing over 20% after adjusting for WBC compositions (Supplementary Tables 8–11), indicating that compositions of WBC were likely to be confounding these associations. For example, one additional drink of wine consumption was associated with a 0.75-year increase of EEAA (*p* = 0.005) in the middle-aged group after adjusting for WBC compositions, while was non-significant without adjustment for WBC compositions (*β* = 0.09, *p* = 0.78) (Supplementary Table 9).

#### Association of long-term average alcohol consumption with epigenetic age acceleration adjusting for long-term average covariate values

In the primary analyses, we utilized cross-sectional covariates (i.e., BMI, PAI, smoke pack-year collected at the same exam when the DNA methylation was measured) in association analyses of long-term average alcohol consumption. In addition, we conducted secondary analyses with long-term average values of covariates in association analyses between long-term average alcohol consumption and EAAs. After adjusting for long-term average values of covariates, we observed mostly consistent associations compared to the primary analyses with cross-sectional covariates (Supplementary Figure 3, Supplementary Table 12). For example, one additional drink of liquor consumption was associated with a 1.49-year increase in PAA (*p* = 4.2e-5) for middle-aged participants after adjusting for long-term average covariates, which was similar to a 1.45-year increase in PAA (*p* = 7.4e-5) in the primary analysis.

### Interaction between sex/smoking status and long-term average alcohol consumption

To investigate whether sex and smoking status modified the association between alcohol consumption and EAAs, we explored the interaction between sex and smoking status with long-term average total alcohol consumption in each age group and the pooled sample. No significant interaction was observed between sex and long-term average total alcohol consumption (Supplementary Table 13). Smoking status did not modify the associations of long-term average total alcohol consumption with most EAAs. The only exception was smoking status modified the association of IEAA with total alcohol consumption in the middle-aged participants and the pooled sample (Supplementary Table 14). One additional drink of total alcohol consumption in former smokers increased 0.73-year in IEAA compared to the increase in non-smokers in the middle-aged group (*p* = 0.004).

### Mediation analysis of epigenetic age acceleration

To investigate whether EAA mediates the association of long-term average alcohol consumption with hypertension, we conducted mediation analyses in each of three age groups and the pooled sample. We found long-term average total alcohol consumption as well as long-term average wine and liquor consumption were associated with higher odds of hypertension in the middle-aged group (Supplementary Table 15). For example, one additional drink of long-term average total alcohol consumption per day was associated with 1.27 times of odds of having hypertension (95% CI: 1.09 to 1.48; *p* = 0.002) in middle-aged participants, adjusting for sex, PAI, education, BMI, smoke pack-year, chronological age, and lab index. Similarly, one additional drink of beer (OR = 1.19, *p* = 0.03), wine (OR = 1.28, *p* = 0.04), and total alcohol consumption (OR = 1.19, *p* = 0.002) was associated with higher odds of hypertension in the pooled sample. Both GAA and PAA were also significantly associated with hypertension adjusting for the same set of covariates in the middle-aged group and the pooled sample (Supplementary Table 15). For example, a one-year increase in GAA and PAA was associated with 1.09 times and 1.05 times of odds of having hypertension in the middle-aged participants, respectively.

With mediation analyses, we found that GAA and PAA displayed mediating effects in most of the associations between long-term average alcohol consumption variables and hypertension in the middle-aged group and the pooled sample (*p* < 0.01) (Supplementary Table 16). For example, in the middle-aged group, 12.62% and 19.30% of associations of hypertension with long-term average wine and liquor consumption were mediated by PAA, respectively. GAA mediated 16.43% of the association between total alcohol consumption and hypertension in middle-aged participants. Similarly, 13.30% and 13.33% of the association of total alcohol consumption with hypertension was mediated through GAA and PAA, separately, in the pooled sample. The largest proportion of mediation in the pooled sample was observed by GAA in the association of liquor consumption with hypertension, that is, 27.55% of the association between liquor consumption and hypertension was mediated by GAA.

## DISCUSSION

In this study, we explored the association of long-term average alcohol consumption with EAA across the whole adulthood in the 3823 FHS participants. We observed long-term average alcohol consumption (i.e., total alcohol, wine, beer, and liquor consumption) were associated with the increase in GAA and PAA in middle-aged and older participants, while no association were observed among young adults. Additionally, the association between long-term average alcohol consumption and EAA appeared to be linear by comparing associations of non-drinkers, at-risk drinkers, and heavy drinkers with light drinkers as the reference. Furthermore, we found that up to 28% of the association between alcohol consumption (total, beer, or liquor) and hypertension was potentially mediated by GAA and PAA in the pooled sample.

Even though several previous studies have investigated associations of alcohol consumption with epigenetic aging, these previous studies have only focused on pooled samples without age stratification [16–18, 24], used small sample sizes (<200) [25, 26], or investigated associations of epigenetic aging with only total alcohol consumption [17, 18, 26]. Nonetheless, after comparing these previous studies with our study, we noticed consistent results on associations of alcohol consumption with GAA and PAA in middle-aged and older participants [17, 18, 24]. For example, a similar result, a higher level of GAA with the mounting number of alcoholic drinks, was also observed in middle-aged and older African Americans and non-Hispanic white women (mean age: 55 and 57 years old) [17, 18]. Of note, we also observed a few inconsistent findings. For example, a lower level of EEAA was associated with greater alcohol intake in a multi-ancestry study where participants with randomly applied treatments were involved [24], while such an association was not observed in our study. Even though it was unclear what factors led to this discrepancy, we suspected that the altered genetic expression and lifestyle factors from clinical trials would modify the association between alcohol consumption and epigenetic aging.

A few studies focused on young adults with large sample sizes to explore epigenetic aging with alcohol consumption. Compared to a previous study, which found alcohol consumption was associated with an increase in GAA in young adults of European and African ancestries [16], we found that there was no significant association between alcohol consumption and GAA in the young adult group in the FHS. Differences might come from different racial compositions in two studies. Our study only included European Americans while the previous study included an approximately equal proportion of participants of European and African ancestries [16]. This previous study reported participants of African ancestry displayed a greater level of GAA than those of European ancestry when they consumed a similar amount of alcohol [16]. In addition, alcohol metabolism and alcohol-related hepatocellular injury are different among racial groups, which may influence epigenetic aging [27, 28].

In this study, differential associations of alcohol consumption with EAAs were observed between three age groups. Younger participants possessed a shorter length of alcohol exposure compared to middle-aged and older participants. The younger age group was also likely to have weaker epigenetic aging due to their younger age compared to the other two age groups. Therefore, associations of alcohol consumption with EAAs are likely to be weaker and require a greater sample size to detect a significant association among young participants if an association exists, compared to those in middle-aged and older groups.

We further noticed that associations varied between five EAAs with the same type of alcoholic beverage. This observation is likely due to the fact that five EAAs were composed of different sets of CpGs. We investigated the correlations between these five EAAs and found that these EAAs were only weakly (Pearson correlation r ≤ 0.3) or moderately correlated with each other (0.3 < Pearson correlation r < 0.6) (Supplementary Figure 4).

We found total alcohol consumption and consumption of each type of alcoholic beverage were associated with a greater increase in GAA and PAA, two EAA metrics derived based on multiple factors in blood chemistry and clinical biomarkers such as those involved in the inflammatory processes [14, 15]. One of the most deleterious effects of ethanol metabolism is oxidative stress [29], which promotes cellular alterations that may activate various transcription factors including growth differentiation factor 15 (GDF-15) [30, 31]. GDF-15 is expressed in multiple tissues and cells (e.g., cardiomyocytes, adipocytes, and macrophages) and is increasingly recognized as an emerging therapeutic target for cardiometabolic disease. Of note, GDF-15 is one of the seven proteins utilized to derive GAA [15]. In this study, the level of DNAm GDF-15 was positively correlated with alcohol consumption in middle-aged and pooled participants (Supplementary Figure 5). PAA is also derived using chronological age and nine clinical biomarkers such as C-reactive protein (CRP), a systematic biomarker of inflammation that plays an important role in the pathology of atherosclerosis [14]. Additionally, CRP level in plasma has previously been associated with alcohol consumption [32–34] and increases with tissue infection, necrosis, and chronic inflammatory conditions [35, 36].

We also observed varied associations of EAAs in their associations with the alcoholic beverage types (i.e., beer, wine, and liquor). However, the effect sizes of associations of five EAAs with three types of alcoholic beverages were, on average, relatively strongly correlated with each other (pair-wise correlation r_beer-wine_ = 0.76; r_beer-liquor_ = 0.88; r_wine-liquor_ = 0.66) (Supplementary Figure 6). Therefore, ethanol content played a major role in the associations of different types of alcoholic beverages with epigenetic aging. Unique components in each type of alcoholic beverage might be related to epigenetic aging through different pathways. For example, resveratrol in wine may explain the deceleration of IEAA in its association with wine consumption among young participants. Furthermore, the cancer-related components (e.g., N-nitrosamines, urethane) and lack of antioxidant compounds in the liquor could account for its stronger associations with EAAs than beer and wine consumption [37, 38]. Unmeasured confounders such as dietary and environmental factors may also lead to differential associations between alcoholic beverages, which warranted further investigations.

We observed up to 28% of the association between long-term average alcohol consumption and hypertension was mediated by GAA or PAA in the FHS. A previous study demonstrated alcohol consumption enhances blood pressure through inhibition of endothelial nitric oxide synthase and promotion of oxidative injury to the endothelium [21]. DNAm at gene promoter regions, catalyzed by DNA methyl transferases, may stimulate proliferation and inflammation in endothelial cells [39]. In addition, ROS in aging endothelial cells may reinforce this DNAm-related damage [40]. In other words, alcohol consumption may promote hypertension via a DNAm-related vicious cycle.

This study has several strengths. We investigated the association of alcohol consumption and different types of alcoholic beverages with EAA metrics in the entire adulthood with a large sample size sample in the well-established, community-based FHS (*n* = 3823). To account for confounding, we adjusted several covariates and lifestyle factors that are associated with alcohol consumption and DNAm. In addition, we also accounted for lab-specific surrogate variables (SVs), which may minimize batch effects and account for unobserved confounding factors. The longitudinal nature of the FHS enabled us to evaluate the association of accumulative alcohol consumption with EAA metrics, which was likely to reduce the recall bias of alcohol consumption [41].

Several limitations are worth noting. First, we only included European Americans in this study, and therefore, the observed associations may not be generalizable to other races and ethnic groups. Additionally, the FHS participants, in general, possess a higher education level than the national average education level (under college: 36.8% in US national average vs. 27.3% in FHS Offspring and Third Generation cohorts) with few young participants under college [42]. Education was an important factor related to alcohol consumption habits (e.g., the amount of alcohol consumed and the type of alcoholic beverages). Therefore, additional studies with large sample sizes are needed to better understand the association between epigenetic aging and alcohol consumption with education stratification. Finally, DNAm was measured using whole blood. Blood connects tissues and the blood-derived epigenetic change may be relayed to other tissues and alter pathophysiological processes in those tissues. Nevertheless, different tissues may display different aging-related DNAm levels [43]. At present, it is not practical to obtain samples from the most metabolically crucial tissues in population studies; future studies may consider collecting and analyzing DNAm levels in such tissues.

In summary, this study investigated alcohol-associated biological aging using EAA metrics in a large FHS sample across the entire adulthood. Our findings may help to understand the role of alcohol-associated biological aging in the development of age-related diseases such as CVD and cancer. Future studies are needed to investigate these associations in large, diverse populations.

## METHODS

### Study participants

**Figure 2:**
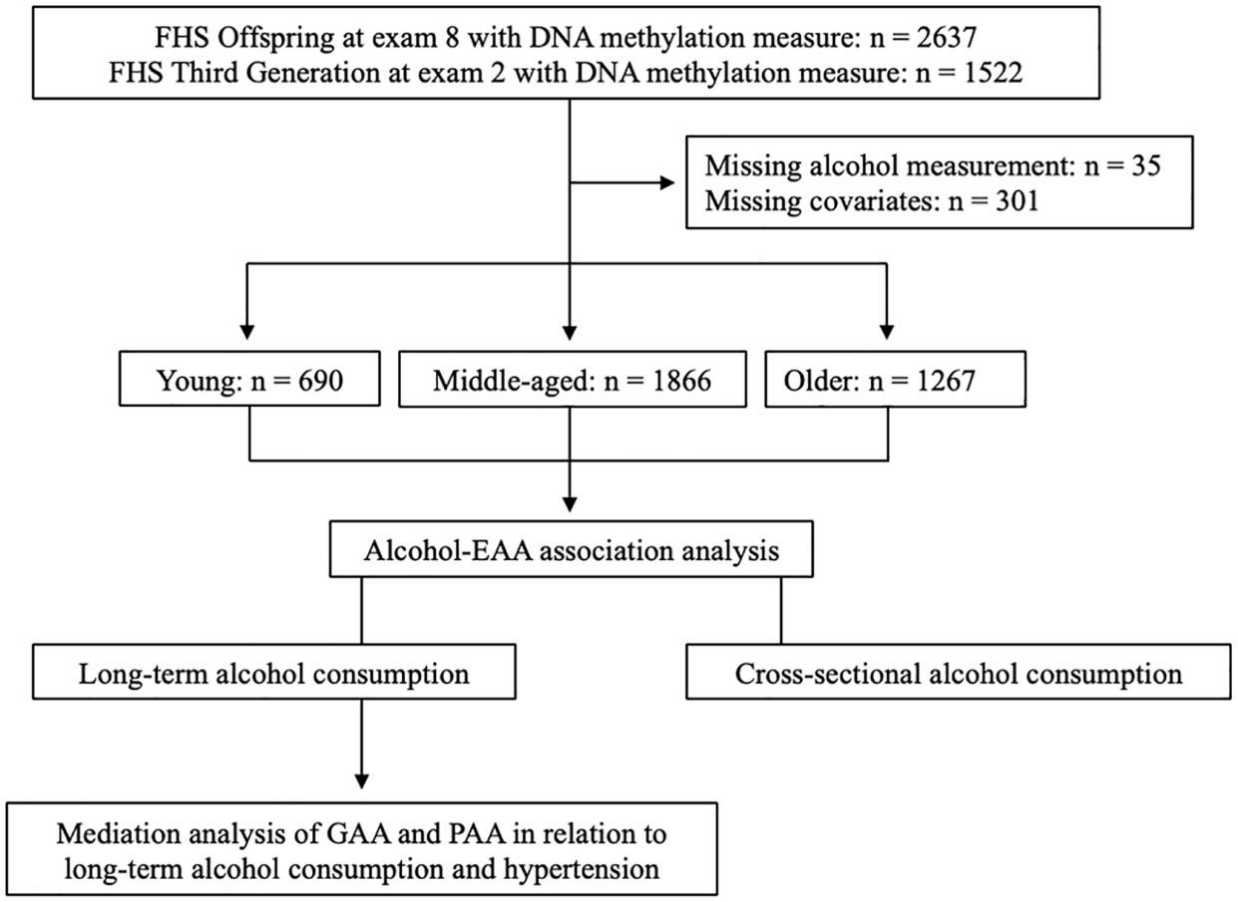
**The flow chart of study design.** We categorized the Framingham Heart Study Offspring and Third Generation participants into three age groups: young (24–44 years, *n* = 690), middle-aged (45–64 years, *n* = 1866), older (65*–*94 years, *n* = 1267). We conducted association analyses of long-term average and cross-sectional alcohol consumption with EAA metrics in each age group. We used the pooled sample to estimate to what extent GAA and PAA mediates the association between alcohol consumption and hypertension.

The Framingham Heart Study (FHS) is a longitudinal cohort study initially established to identify cardiovascular risk factors. From 1948 to 1953, the FHS recruited 5209 residents of Framingham, Massachusetts into the Original cohort [44]. Children of the Original cohort and the spouses of the children (*n* = 5124) were enrolled in the Offspring cohort since 1971 [45]. The Third Generation cohort was enrolled since 2002 (*n* = 4095) [46]. Starting from enrollment, Offspring and Third Generation participants underwent regular health examinations every 4–8 years to collect demographic information and cardiovascular risk factors [47]. The Offspring cohort has undergone nine examinations and the Third Generation cohort has undergone three examinations. Peripheral blood samples have been collected from study participants at each examination. This study utilized participants with DNAm measurements from the Offspring participants at exam 8 and the Third Generation participants at exam 2. Because this study used longitudinal alcohol measures, we removed Offspring participants without alcohol consumption measurements at exam 1 and exam 8, then retained those who had at least one alcohol consumption measurement between exam 2 and exam 7. All Third Generation participants with DNAm measurement had alcohol consumption measurements at both exam 1 and exam 2. In addition, we excluded participants with missing values in any of the covariates. Finally, 2321 participants in the Offspring cohort and 1502 participants in the Third Generation cohort remained for subsequent analyses (Figure 2). Among the entire study participants (women 53.8%), ages ranged from 24 to 94 years with an average age of 58.1 years when DNAm was measured.

### DNA methylation measurement in whole blood and quality control

DNAm profiling and quality control procedures have been described previously [10]. Briefly, 2846 participants from the Offspring cohort attending exam 8 and 1549 participants from the Third Generation cohort attending exam 2 received DNAm measurement using the Infinium HumanMethylation 450K BeadChip array (Illumina, Inc., San Diego, CA, USA). Genomic DNA was extracted from whole blood samples collected at routine exams. DNAm profiling was conducted by bisulfite conversion, followed by whole genome amplification, fragmentation, array hybridization, and single-base pair extension (the manufacturer’s protocols) [48]. The DNAm levels in Offspring participants were measured in two separate laboratories (*n* = 576 and 2270) and the DNAm levels in Third Generation participants were measured by the third laboratory in Illumina. Several quality control procedures were applied separately to each batch of DNAm data. Briefly, we removed cross-reactive probes that mapped to multiple locations at the CpG level [49]. We also removed low-quality probes with a high missing rate (> 20%), with detection p > 0.01, and with single nucleotide polymorphisms (SNPs) at CpG sites or ≤ 10 bp of single base extension [49, 50]. At the participant level, we excluded individuals if they had a missing rate > 1% or were outliers according to multi-dimensional scaling (MDS) analysis [51] and with a poor match to the 65 SNP genotypes on the Infinium HumanMethylation 450K BeadChip array.

### Epigenetic age acceleration

The primary outcome variables were age accelerations based on PhenoAge [14] and GrimAge [15], which are the second generation of epigenetic age variables estimating biological age based on aging-related phenotypic biomarkers [52]. Biological age predicts chronological age with clinical biomarkers and reveals differences in biological aging levels by their significant associations with disease-specific morbidity and mortality [53, 54]. PhenoAge was calculated from 513 CpGs, reflecting morbidity and mortality of diseases [14]. GrimAge specially considered smoking pack-year-related CpGs and was calculated from 1030 CpGs [15]. As secondary analyses, three additional EAA variables based on Horvath’s age [11], Hannum’s age [12], and skin and blood clock [13] were used as outcome variables. Horvath’s age utilized 353 CpGs based on DNAm measurements from multi-tissues, and the corresponding age acceleration reflects IEAA in cells [11, 55]. Hannum’s age utilized 71 CpGs from blood samples, and its age acceleration, EEAA, tracked aging-related changes in blood cell components compared to IEAA [12, 55]. Skin and blood clock and its age acceleration (EAASkinBlood) focused on skin and blood samples and were estimated from 391 CpGs [13]. We used Horvath’s online calculators to compute lab-specific age accelerations for two primary and three secondary epigenetic age variables [11]. EAA was estimated as the residual from a linear model of chronological age regressed on each of the epigenetic age metrics. Of note, the epigenetic age variables were highly correlated with chronological age while EAA metrics were not correlated with chronological age (Supplementary Figures 4, 7).

### Alcohol consumption measurement

#### Continuous alcohol consumption variable

Alcohol consumption was measured at each follow-up exam in Offspring and Third Generation cohorts. The FHS participants were invited to answer questions from a questionnaire that included several questions regarding the consumption of alcoholic beverages [56]. These questions included “Number of glasses you drink per week over the past year” for beer (12 oz/glass), wine (4 oz/glass from white or red wine), and liquor (1.25 oz/glass). The total alcohol consumption per day was created by summing up beer, wine, and liquor consumption and divided by seven days. Consumption of each type of alcoholic beverage was defined as the total consumption of that type of alcoholic beverage per day. To capture the association of long-term average alcohol consumption with EAA, our main predictor was the average of beer/wine/liquor/total alcohol consumption from exam 1 to exam 8 for the Offspring participants and from exam 1 to exam 2 for the Third Generation participants. The cross-sectional beer/wine/liquor/total alcohol consumption was measured at exam 8 and exam 2 for the Offspring participants and the Third Generation participants separately.

#### Categorical alcohol consumption variable

To determine whether alcohol consumption has a linear relationship with EAA, participants were divided into four groups based on the long-term average total alcohol consumption. Details for defining drinking categories were described in the previous study [10]. Non-drinkers were participants with an average total alcohol consumption equal to zero. Light drinkers included women who consumed less than 1 drink per day and men who consumed less than 2 drinks per day. Women who consumed 1–2 drinks per day and men who consumed 2–3 drinks per day were declared at-risk drinkers. Women who consumed more than 2 drinks per day and men who consumed more than 3 drinks per day were declared as heavy drinkers.

#### Binge drinking variable

In the questionnaire, recent binge drinking was assessed with the questions “The maximum number of drinks in 24 hours in the last month” and “How many drinks do you have on a typical day over the last year” for the total alcohol consumption. We defined women consuming over 4 drinks per day and men consuming over 5 drinks per day as having binge drinking. The number of drinking days was also considered for binge drinking with the question “On average how many days per week did you drink over the past year” for the total alcohol consumption. Participants with less than 2 alcohol-free days per week were additionally included in the recent binge drinking group.

### Hypertension

To explore whether EAA metrics mediated the association between alcohol consumption and prevalent hypertension, we defined hypertension as participants with systolic blood pressure (SBP) ≥ 130 mmHg or diastolic blood pressure (DBP) ≥ 80 mmHg or any medication to lower blood pressure in Offspring participants at exam 8 and Third Generation participants at exam 2 [57].

### Covariates

In order to control for confounding, the following covariates at exam 8 (Offspring) and at exam 2 (Third Generation) were considered: sex, PAI, education, BMI, chronological age, smoking pack-year, lab, and surrogate variables (SVs). The PAI score combined weighted hours of slight, moderate, and heavy activity [58] to capture the total energy expenditure from body movements. The hours to conduct the slight, moderate, and heavy activities were recorded by the questions “the number of hours with slight/moderate/heavy activities for a typical day.” We modified the metabolic equivalent score (MET-score) formula used in the International Physical Activity Questionnaire (IPAQ) to calculate PAI in this study [59]:


PAI=3.3×slight hours+4×moderate hours+8×heavy hours

We categorized education into three levels: without a college degree, some college degree, and a four-year college degree or above. Smoking status was categorized into three levels: non-smokers, former smokers, and current smokers. Pack-year of smoking variable measured both smoking quantity and smoking duration by multiplying the number of cigarette packs per day by years of smoking [60]. The number of cigarettes consumed per day was assessed with the following question “How many cigarettes do you smoke per day now” for each participant in the Offspring and the Third Generation cohorts and transformed into the number of packs per day by assuming 20 cigarettes in each pack. Age at initiation and end of smoking were additionally collected to capture the total years of smoking.

### Statistical analysis

For primary analyses, the participants (2321 Offspring participants and 1502 Third Generation participants) were divided into three age groups: young (24–44 years, n_young_ = 690), middle-aged (45–64 years, n_middle-aged_ = 1866), and older age (> 64 years, n_older_ = 1267) groups. We formally assessed if the age group modified the association between long-term average alcohol consumption and EAAs in participants of three age groups. For all analyses, lab-specific SVs were imputed to account for unobserved confounding effects by the package “sva” [61]. Lab-specific EAA residuals were obtained by regressing EAA metrics on SVs and were used as outcome variables. In primary analyses, we used linear regression models to quantify the association of long-term average alcohol consumption (i.e., total alcohol consumption and each type of alcoholic beverage consumption) with EAA residuals in each of the three age groups. All regression models adjusted for covariates including sex, PAI, education level, BMI, smoking pack-year, chronological age, and lab index variable. To investigate whether the association between alcohol consumption and EAA residuals was linear, we compared the mean differences in EAA residual variable between drinking categories, i.e., non-drinkers, at-risk drinkers, and heavy drinkers, versus light drinkers as the reference group (the drinking group with the largest number of participants), adjusting for the same set of covariates.

We conducted several secondary analyses. First, we investigated the associations of cross-sectional alcohol consumption and recent binge drinking with EAA residuals (outcome variables) in each of the three age groups. We conducted association analyses of long-term average and cross-sectional alcohol consumption with EAA residuals in the pooled sample (i.e., pooling participants across the three age groups), adjusting for the same set of covariates. We conducted secondary association analyses, with additional adjustments for imputed white blood cell compositions (including CD8+ T cells, CD4+ T cells, natural killer cells, B cells, monocytes, and granulocytes) based on the Houseman’s method [10, 62]. In addition, we conducted secondary association analyses of long-term average alcohol consumption with EAAs adjusting for long-term average values of covariates (i.e., average BMI, PAI, and smoke pack-year). We also evaluated whether sex and smoking status modified the association between long-term average total alcohol consumption and EAA residuals in each age group and the pooled sample, by including an interaction term (i.e., sex × alcohol or smoking × alcohol) in the regression model. To account for multiple testing, we used *p* < 0.01 (= 0.05/5) for significance; here we considered accounting for two primary and three secondary EAA measures.

Previous studies have shown that alcohol consumption increases the odds of hypertension [21, 56, 63]. We, therefore, examined the association of long-term average alcohol consumption and residuals of GAA and PAA with hypertension utilizing the mediation analysis “PROC CAUSALMED” in SAS. All statistical analyses were performed with R software (version 4.1.1) and SAS software (version 9.3).

## Supplementary Materials

Supplementary Figures

Supplementary Tables
