# Supplementary material for: Alcohol consumption and epigenetic age acceleration across human adulthood
Source: Aging (Albany NY). 2023 Oct 26;15(20):10938–71. doi: 10.18632/aging.205153 (PMC10637803; doi:10.18632/aging.205153)
Supplement: Supplementary Figures [file aging-15-205153-s001.pdf]

SUPPLEMENTARY FIGURES

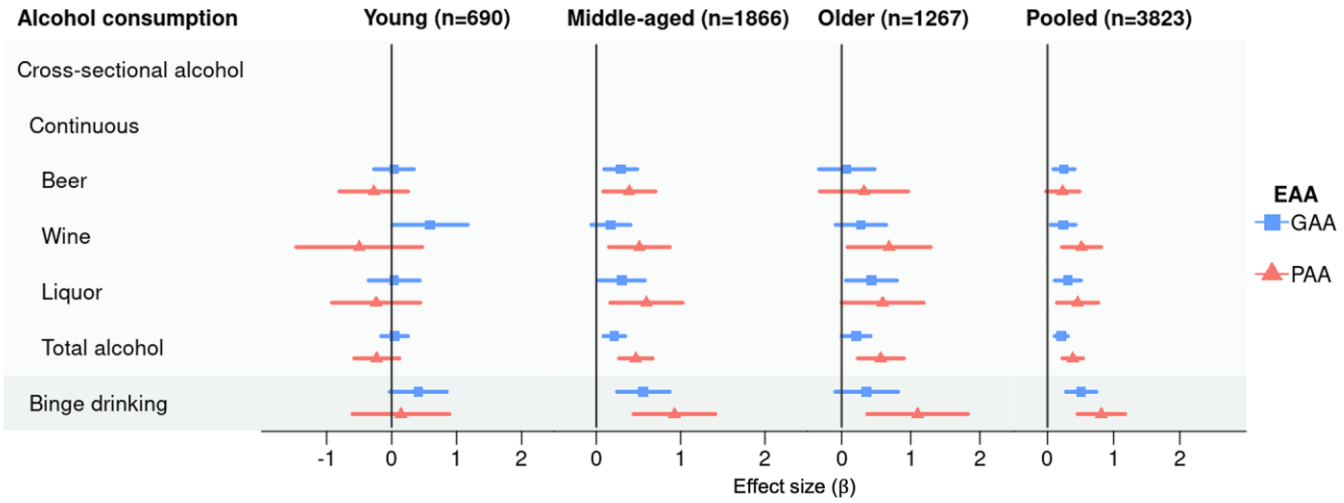

Supplementary Figure 1. Association analyses between cross-sectional alcohol consumption and EAAs in each age groups and in pooled samples in the Framingham Heart Study. Age groups: young (24-44 years), middle-aged (45-64 years), older (65–94 years). The x-axis represented the effect size of alcohol consumption on GAA or PAA. Covariates included sex, physical activity score, education level, BMI, smoke pack-year, chronological age, and lab. The cross-sectional alcohol consumption represented the latest cross-sectional total alcohol consumption. The binge drinking represented the recent binge drinking based the cross-sectional alcohol consumption and number of free-alcohol days per week. Abbreviations: GAA: GrimAge acceleration; PAA: PhenoAge acceleration. Effect sizes and *p*-values can be found in Supplementary Tables 5, 7.

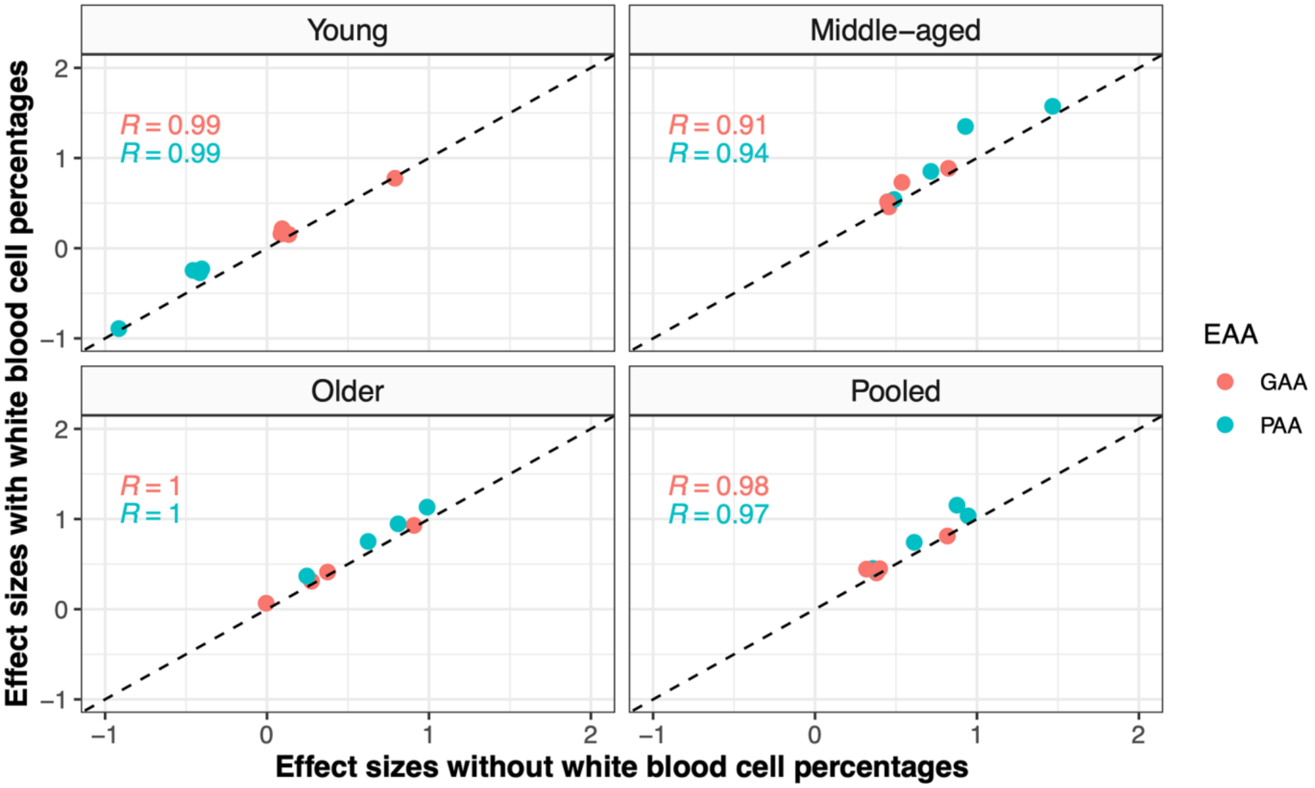

Supplementary Figure 2. Comparison of effect sizes between models with and without adjustment of white blood cells. This figure indicated the comparison of effect sizes between the primary analysis and the secondary analysis with adjustment of white blood cell compositions. The dot line in each plot was the reference line: y = x. Each facet contained eight dots, where four red dots represented effect sizes from associations of GAA with three types of alcoholic beverage (i.e., beer, wine, and liquor) and total alcohol. Similarly, four blue dots in each facet represented effect sizes from associations of PAA with three alcoholic beverage types and total alcohol. Models without white blood cell compositions were primary analyses to explore associations between alcohol consumption and EAAs. Covariates included sex, education level, chronological age, lab, BMI, physical activity score, and smoke pack-year. Models with white blood cell compositions were secondary analyses by additionally adjusting for percentages of white blood cells. Effect sizes in all models represented the change of EAA with one additional drink of long-term average alcohol consumption in each age group and the pooled sample. Abbreviations: GAA: GrimAge acceleration; PAA: PhenoAge acceleration.

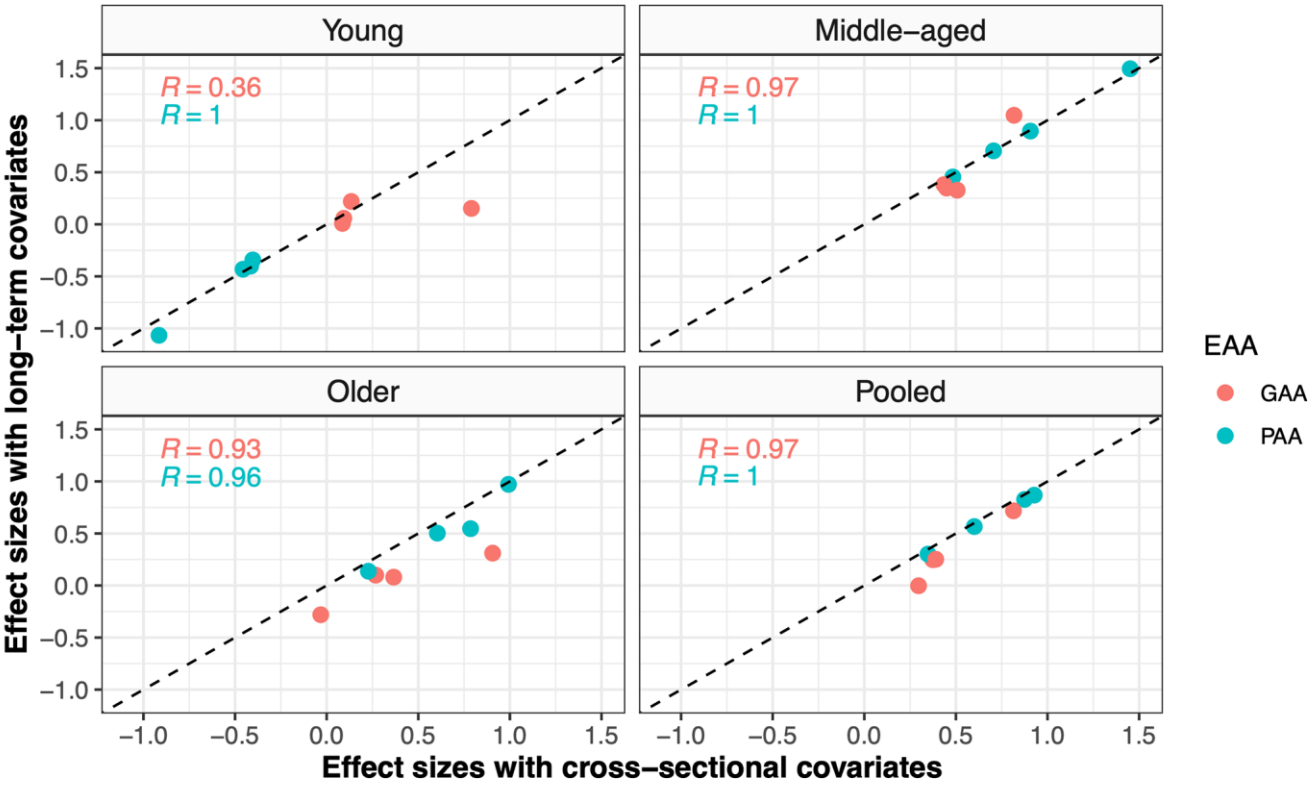

Supplementary Figure 3. Comparison of effect sizes between models with cross-sectional covariates and long-term covariates. This figure indicated the comparison of effect sizes between the primary analysis and the secondary analysis with long-term covariates. The dot line in each plot was the reference line: y = x. Each facet contained eight dots, where four red dots represented effect sizes from associations of GAA with three types of alcoholic beverage (i.e., beer, wine, and liquor) and total alcohol. Similarly, four blue dots in each facet represented effect sizes from associations of PAA with three alcoholic beverage types and total alcohol. Models with cross-sectional covariates were primary analyses to explore associations between alcohol consumption and EAAs. Covariates included sex, education level, chronological age, lab, BMI, physical activity score, and smoke pack-year. Models with long-term covariates were secondary analyses by replacing three cross-sectional covariates with long-term average values (i.e., BMI, physical activity score, and smoke pack-year). Effect sizes in all models represented the change of EAA with one additional drink of long-term average alcohol consumption in each age group and the pooled sample. Abbreviations: GAA: GrimAge acceleration; PAA: PhenoAge acceleration.

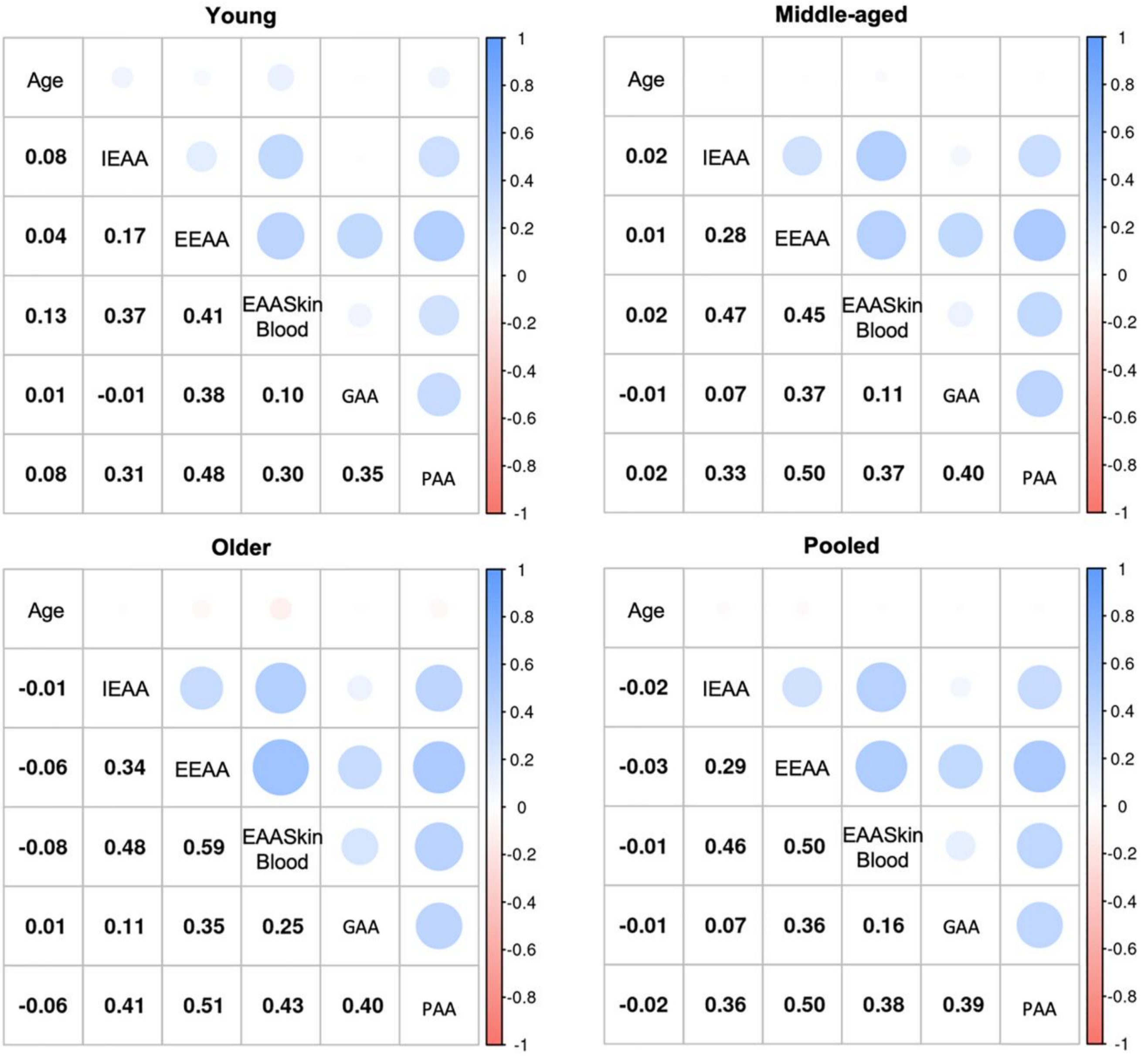

Supplementary Figure 4. Correlation between chronological age and EAAs. This figure indicated the correlation between chronological age and each epigenetic age acceleration (EAA) in each age group and the pooled sample. Abbreviations: IEAA: intrinsic epigenetic age acceleration; EEAA: extrinsic epigenetic age acceleration; EAASkinBlood: epigenetic age acceleration with skin and blood clock; GAA: GrimAge acceleration; PAA: PhenoAge acceleration.

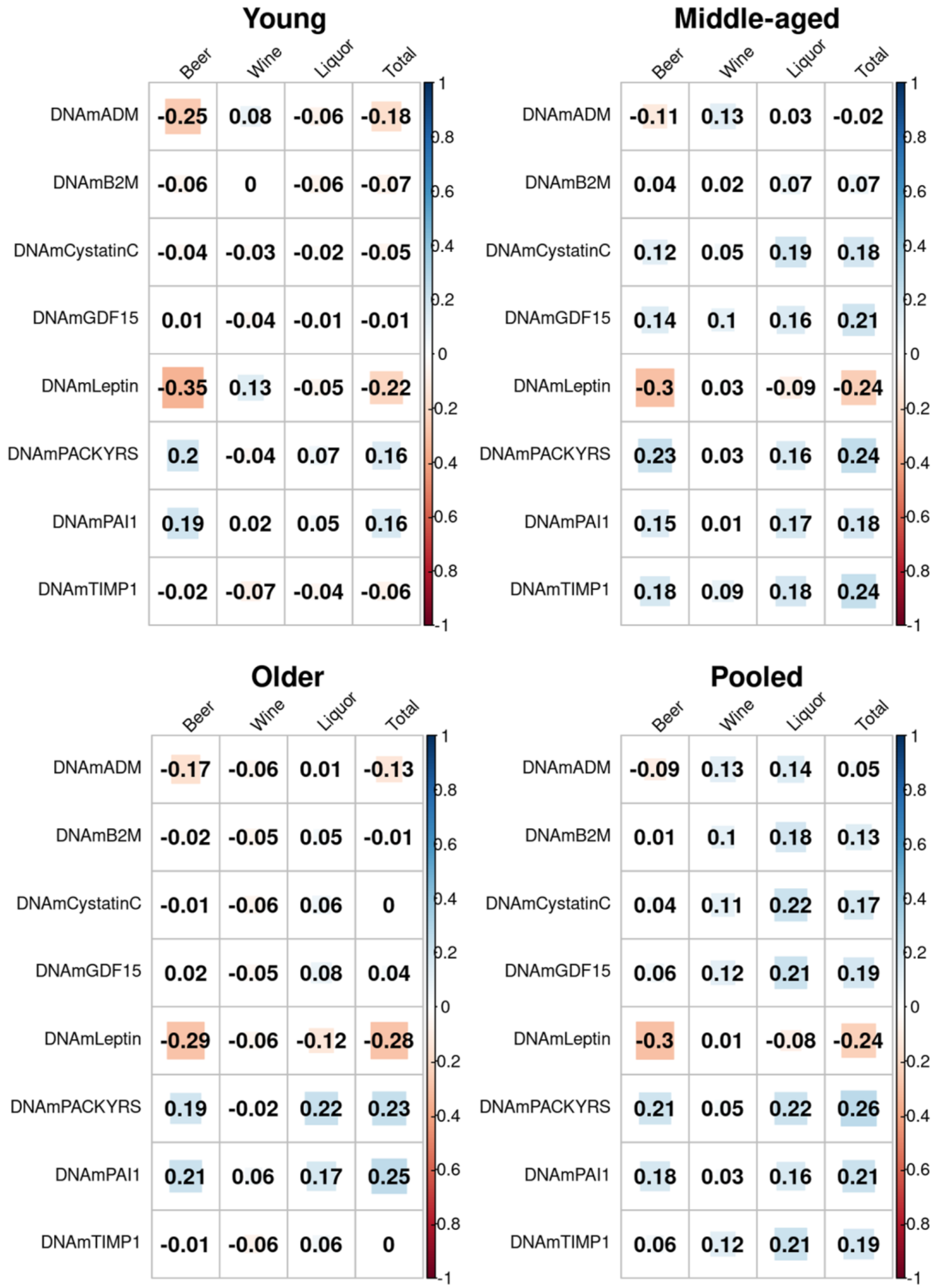

Supplementary Figure 5. Correlation between long-term average alcohol consumption and DNAm-based biomarkers from GAA. This figure indicated the correlation between long-term average alcohol consumption (beer, wine, liquor, and total alcohol consumption) and DNAm-based biomarkers from GAA in young, middle-aged, older, and pooled participants. Abbreviations: ADM: adrenomedullin; B2M: beta 2 microglobulin; GDF 15: growth differentiation factor 15; PCKTRS: smoking pack-year; PAI1: plasminogen activator inhibitor 1; TIMP1: tissue inhibitor metalloproteinases 1.

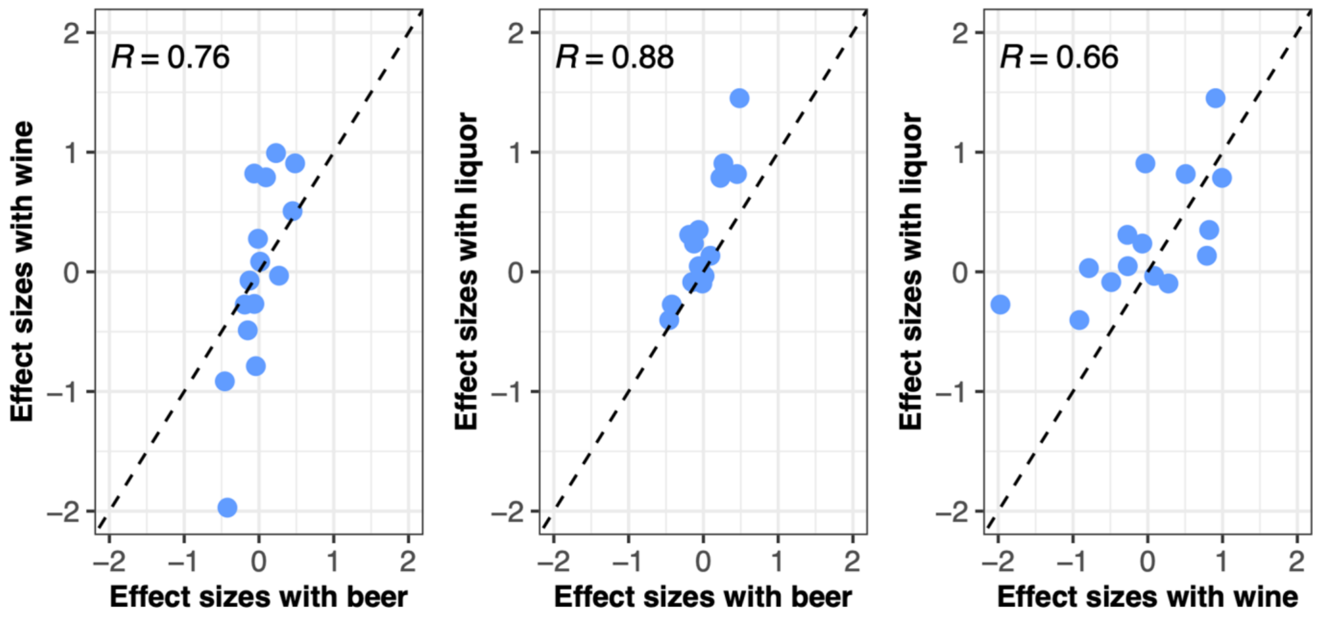

Supplementary Figure 6. Comparison of associations of alcohol consumption with EAAs between alcoholic beverage types. This figure indicated the comparison of effect sizes between three types of alcoholic beverages in the primary analyses. The dot line in each plot was the reference line: y = x. Each facet contained fifteen dots, representing effect sizes in association between one type of alcoholic beverage (i.e., beer, wine, liquor) and five EAAs in three age groups. Effect sizes in all models represented the change of EAA with one additional drink of long-term average alcohol consumption in each age group. All models were adjusted for sex, education level, chronological age, lab, BMI, physical activity score, and smoke pack-year.

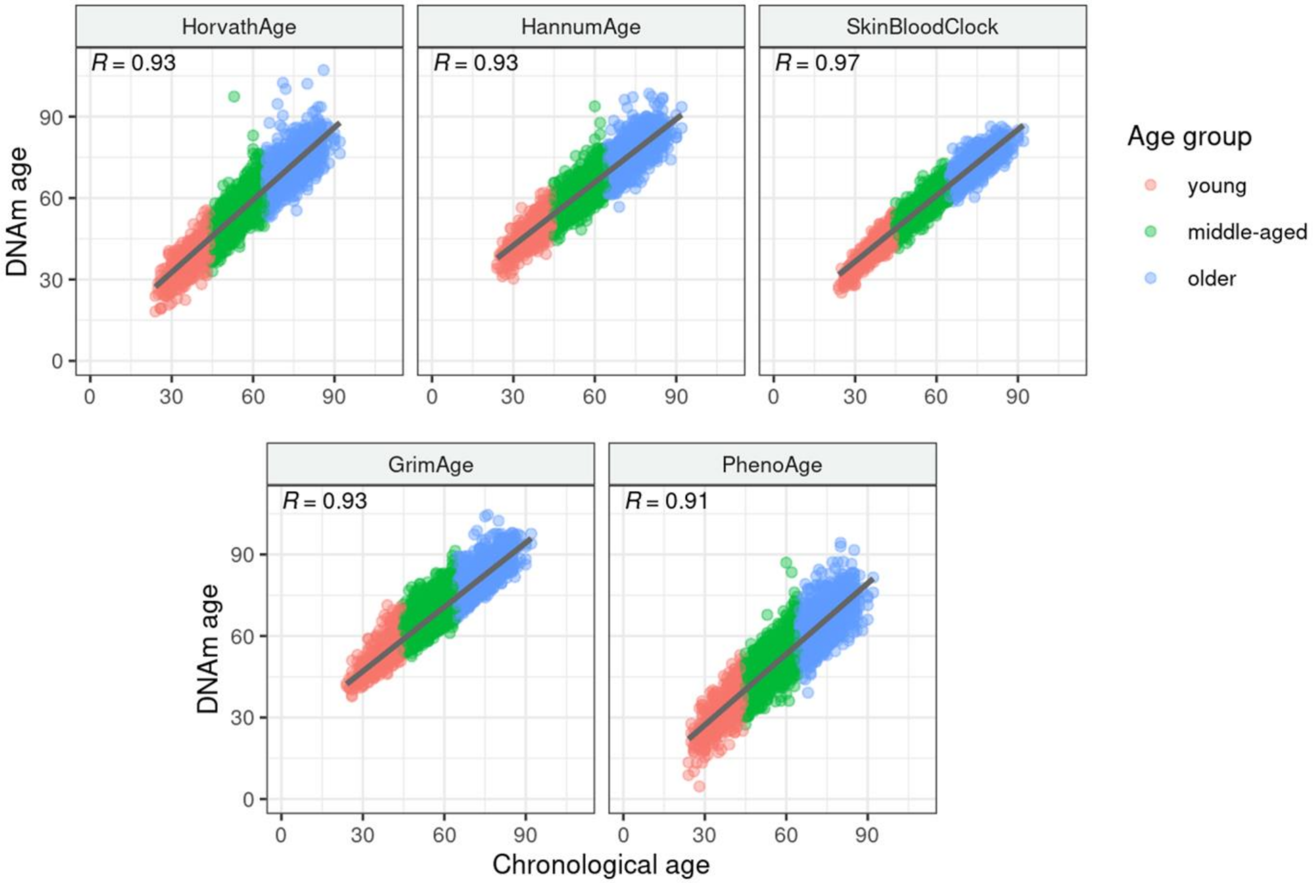

Supplementary Figure 7. Correlation between chronological age and DNAm ages. This figure indicated the correlation between chronological age and each DNAm age in the pooled sample. Red dots represented young participants, green dots represented middle-aged participants, and blue dots represented older participants. Gray lines represented regression line between each DNAm age and chronological age in the pooled sample.
