# Supplementary material for: Alcohol consumption and epigenetic age acceleration across human adulthood
Source: Aging (Albany NY). 2023 Oct 26;15(20):10938–71. doi: 10.18632/aging.205153 (PMC10637803; doi:10.18632/aging.205153)
Supplement: Supplementary Tables [file aging-15-205153-s002.pdf]

SUPPLEMENTARY TABLES

Supplementary Table 1. Interaction between long-term total alcohol consumptions and age groups in the pooled analysis.

	β	*p*	LCI	UCI	Overall *p*
IEAA					
Total alcohol consumption×age group					
Young			ref		
Middle-aged	0.53	0.09	−0.08	1.14	0.23
Older	0.50	0.11	−0.11	1.12	
EEAA					
Total alcohol consumption×age group					
Young			ref		
Middle-aged	0.11	0.76	−0.57	0.78	0.95
Older	0.10	0.78	−0.58	0.78	
EAASkinBlood					
Total alcohol consumption×age group					
Young			ref		
Middle-aged	0.21	0.29	−0.17	0.59	0.47
Older	0.24	0.22	−0.14	0.63	
GAA					
Total alcohol consumption×age group					
Young			ref		
Middle-aged	0.23	0.36	−0.26	0.71	0.60
Older	0.14	0.58	−0.35	0.63	
PAA					
Total alcohol consumption×age group					
Young			ref		
Middle-aged	1.43	2.35E-04	0.67	2.19	9.40E-04
Older	1.36	5.13E-04	0.59	2.12	

All associations were adjusted for sex, physical activity index, education level, BMI, smoke pack-year, chronological age, age groups, and lab with the young-age group (22–44 years) as the reference. The beta (β) coefficients represented the increase in EAA with one additional drink of long-term total alcohol consumption in middle-aged (45–64 years) or older (≥ 65 years) participants, compared to the young-age participants. Overall *p*, *p* value of the interaction term (alcohol*age group). Abbreviations: LCI: low confidence interval; UCI: upper confidence interval; IEAA: intrinsic epigenetic age acceleration; EEAA: extrinsic epigenetic age acceleration; EAASkinBlood: epigenetic age acceleration with skin and blood clock; GAA: GrimAge acceleration; PAA: PhenoAge acceleration.

Supplementary Table 2. Association between long-term average alcohol consumption and EAAs.

		Young		Middle-aged		Older		Pooled	
		β	*p*	β	*p*	β	*p*	β	*p*
Beer	IEAA	−0.42	0.30	−0.13	0.44	−0.19	0.37	−0.17	0.16
	EEAA	−0.06	0.90	0.02	0.93	−0.15	0.51	−0.05	0.72
	EAASkinBlood	−0.04	0.88	−0.01	0.89	−0.06	0.63	−0.02	0.79
	GAA	0.09	0.76	0.45	5.2E-04	0.27	0.10	0.37	1.2E-04
	PAA	−0.46	0.39	0.48	0.02	0.23	0.38	0.35	0.02
Wine	IEAA	−1.97	0.006	−0.07	0.79	−0.27	0.43	−0.25	0.21
	EEAA	0.82	0.33	0.08	0.78	−0.49	0.20	−0.07	0.75
	EAASkinBlood	−0.79	0.09	0.28	0.11	−0.27	0.19	0.01	0.94
	GAA	0.79	0.15	0.51	0.02	−0.03	0.90	0.30	0.06
	PAA	−0.92	0.32	0.91	0.008	0.99	0.02	0.87	5.0E-04
Liquor	IEAA	−0.27	0.62	0.24	0.42	0.31	0.28	0.21	0.25
	EEAA	0.35	0.58	−0.03	0.92	−0.09	0.78	−0.05	0.82
	EAASkinBlood	0.03	0.93	−0.10	0.60	0.05	0.78	−0.01	0.93
	GAA	0.13	0.74	0.82	4.8E-04	0.91	2.5E-05	0.81	2.5E-08
	PAA	−0.40	0.56	1.45	7.4E-05	0.79	0.02	0.93	5.8E-05
Total alcohol	IEAA	−0.51	0.06	−0.02	0.89	0.01	0.95	−0.05	0.57
	EEAA	0.06	0.84	−0.08	0.56	−0.11	0.49	−0.07	0.47
	EAASkinBlood	−0.08	0.65	0.01	0.91	−0.03	0.76	−0.002	0.97
	GAA	0.09	0.67	0.43	5.4E-06	0.37	0.001	0.39	1.3E-08
	PAA	−0.42	0.23	0.71	2.1E-06	0.60	7.5E-04	0.60	2.3E-08

All associations were adjusted for sex, physical activity score, education level, BMI, smoke pack-year, chronological age, and lab. The β coefficients represented the increase in EAA with one additional drink of long-term average alcohol consumption in each group. Abbreviations: IEAA: intrinsic epigenetic age acceleration; EEAA: extrinsic epigenetic age acceleration; EAASkinBlood: epigenetic age acceleration with skin and blood clock; GAA: GrimAge acceleration; PAA: PhenoAge acceleration.

Supplementary Table 3. Association between drinking categories and EAAs.

		Young		Middle-aged		Older		Pooled	
		β	*p*	β	*p*	β	*p*	β	*p*
IEAA	Light drinker	ref							
	Non-drinker	0.01	0.98	0.42	0.20	−0.08	0.88	0.18	0.46
	At-risk drinker	−0.55	0.55	0.39	0.24	−0.66	0.10	−0.14	0.56
	Heavy drinker	−1.27	0.44	0.19	0.71	0.33	0.55	0.20	0.57
EEAA	Light drinker	ref							
	Non-drinker	−0.34	0.51	−0.02	0.96	−0.61	0.32	−0.23	0.40
	At-risk drinker	−0.78	0.47	0.02	0.95	−0.30	0.48	−0.16	0.55
	Heavy drinker	2.79	0.15	0.18	0.75	−0.17	0.78	0.10	0.79
EAA SkinBlood	Light drinker	ref							
	Non-drinker	0.06	0.84	−0.12	0.58	−0.52	0.13	−0.22	0.15
	At-risk drinker	−0.12	0.85	0.10	0.63	−0.36	0.13	−0.12	0.43
	Heavy drinker	0.92	0.39	0.30	0.35	0.18	0.59	0.31	0.17
GAA	Light drinker	ref							
	Non-drinker	−0.44	0.19	0.30	0.25	−0.53	0.23	−0.09	0.66

	At-risk drinker	0.37	0.60	0.70	0.007	0.30	0.33	0.47	0.01
	Heavy drinker	−1.66	0.18	2.17	8.0E-08	1.26	0.004	1.54	7.2E-08
PAA	Light drinker	ref							
	Non-drinker	−0.69	0.23	0.08	0.85	−0.10	0.88	−0.16	0.60
	At-risk drinker	0.28	0.81	1.52	1.6E-04	1.16	0.02	1.27	2.1E-05
	Heavy drinker	−3.66	0.08	2.54	5.4E-05	2.07	0.003	2.12	2.2E-06

All associations were adjusted for sex, physical activity index, education level, BMI, smoke pack-year, chronological age, and lab. The β coefficients represented the increase of EAA in non-drinkers, at-risk drinkers, or heavy drinkers, compared to the light drinkers in each group. Non-drinkers were participants who had long-term average alcohol consumption equal to zero; light drinkers were defined as less than 1 drink per day for women and less than 2 drinks per day for men; at risk drinkers were defined as 1–2 drinks per day for women and 2–3 drinks per day for men; heavy drinkers were defined as more than 2 drinks per day for women and more than 3 drinks per day for men. Abbreviations: IEAA: intrinsic epigenetic age acceleration; EEAA: extrinsic epigenetic age acceleration; EAASkinBlood: epigenetic age acceleration with skin and blood clock; GAA: GrimAge acceleration; PAA: PhenoAge acceleration.

Supplementary Table 4. Participant characteristics of cross-sectional alcohol consumption in age groups.

Variable<br>Median (quantile) or *n* (%)	Young	Middle-aged	Older	*p*
Cross-sectional consumption[1]				
Continuous, drinks/day				
Beer	0.11 (0, 0.43)	0 (0, 0.29)	0 (0, 0.04)	<0.001
Wine	0.11 (0, 0.29)	0.07 (0, 0.43)	0.04 (0, 0.43)	<0.001
Liquor	0 (0, 0.07)	0 (0, 0.11)	0 (0, 0.04)	0.22
Total	0.43 (0.14, 0.96)	0.43 (0.04, 1.07)	0.14 (0, 1.00)	<0.001
Binge drinking	274 (39.71%)	583 (31.24%)	310 (24.47%)	<0.001

Median with quantiles was provided for continuous alcohol consumption variables. [1]The cross-sectional alcohol consumption represented the latest cross-sectional total alcohol consumption. The binge drinking represented the recent binge drinking based the cross-sectional alcohol consumption and number of free-alcohol days per week.

Supplementary Table 5. Association between cross-sectional alcohol consumption and EAAs.

		Young		Middle-aged		Older		Pooled	
		β	*p*	β	*p*	β	*p*	β	*p*
Beer	IEAA	−0.26	0.21	−0.01	0.96	−0.42	0.11	−0.12	0.22
	EEAA	−0.06	0.81	0.15	0.28	−0.31	0.29	0.01	0.94
	EAASkinBlood	−0.02	0.89	−0.02	0.85	−0.12	0.45	−0.05	0.43
	GAA	0.04	0.82	0.29	0.004	0.07	0.72	0.25	0.002
	PAA	−0.28	0.31	0.39	0.01	0.32	0.32	0.23	0.07
Wine	IEAA	−1.11	0.004	−0.13	0.38	0.02	0.93	−0.15	0.20
	EEAA	0.41	0.37	0.13	0.42	−0.19	0.48	0.09	0.52
	EAASkinBlood	−0.50	0.05	0.10	0.26	−0.10	0.49	0.02	0.77
	GAA	0.59	0.05	0.17	0.14	0.28	0.14	0.24	0.01
	PAA	−0.50	0.31	0.51	0.006	0.69	0.02	0.52	6.5E-04
Liquor	IEAA	−0.15	0.58	0.15	0.38	−0.01	0.97	0.04	0.73
	EEAA	0.17	0.59	−0.04	0.83	0.01	0.96	0.01	0.96
	SkinBlood	0.01	0.94	−0.12	0.30	0.02	0.91	−0.05	0.50

	GAA	0.04	0.85	0.30	0.03	0.43	0.02	0.31	0.002
	PAA	−0.24	0.49	0.59	0.007	0.59	0.05	0.46	0.004
Total alcohol	IEAA	−0.27	0.05	−3.5E-04	1.00	−0.07	0.61	−0.06	0.33
	EEAA	0.03	0.85	0.05	0.60	−0.14	0.33	−9.5E-04	0.99
	EAASkinBlood	−0.04	0.65	−0.02	0.68	−0.07	0.38	−0.04	0.31
	GAA	0.05	0.66	0.21	0.001	0.21	0.05	0.21	4.8E-05
	PAA	−0.23	0.20	0.47	4.0E-06	0.57	9.1E-04	0.38	1.6E-06

All associations were adjusted for sex, physical activity index, education level, BMI, smoke pack-year, chronological age, and lab. The β coefficients represented the increase in EAA with one additional drink of cross-sectional alcohol consumption in each group. Abbreviations: IEAA: intrinsic epigenetic age acceleration; EEAA: extrinsic epigenetic age acceleration; EAASkinBlood: epigenetic age acceleration with skin and blood clock; GAA: GrimAge acceleration; PAA: PhenoAge acceleration.

Supplementary Table 6. Overlap between drinking category and binge drinking.

	Non-drinker	Light drinker	At-risk drinker	Heavy drinker
Binge	2 (0.63%)	789 (26.01%)	253 (76.20%)	123 (87.23%)
Non-binge	315 (99.37%)	2244 (73.99%)	79 (23.80%)	18 (12.77%)

All statistics represented number of participants overlapped by the drinking category and the recent binge drinking. Four drinking categories were defined with long-term consumption. Non-drinkers were participants who had long-term average alcohol consumption equal to zero; light drinkers were defined as less than 1 drink per day for women and less than 2 drinks per day for men; at risk drinkers were defined as 1–2 drinks per day for women and 2–3 drinks per day for men; heavy drinkers were defined as more than 2 drinks per day for women and more than 3 drinks per day for men. The cross-sectional alcohol consumption represented the latest cross-sectional total alcohol consumption. The binge drinking represented the recent binge drinking based the cross-sectional alcohol consumption and number of free-alcohol days per week.

Supplementary Table 7. Association between recent binge drinking and EAAs.

	Young		Middle-aged		Older		Total	
	β	*p*	β	*p*	β	*p*	β	*p*
IEAA	−0.13	0.66	−0.09	0.66	−0.07	0.83	−0.07	0.61
EEAA	0.56	0.11	−0.28	0.22	−0.67	0.04	−0.22	0.17
EAASkinBlood	0.18	0.34	−0.22	0.08	−0.28	0.11	−0.16	0.08
GAA	0.41	0.07	0.56	4.5E-04	0.36	0.12	0.51	1.3E-05
PAA	0.14	0.70	0.93	1.8E-04	1.10	0.003	0.81	9.1E-06

All associations were adjusted for sex, physical activity index, education level, BMI, smoke pack-year, chronological age, and lab. The β coefficients represented the increase of EAA in participants with recent binge drinking, compared to those without recent binge drinking in each group. Abbreviations: IEAA: intrinsic epigenetic age acceleration; EEAA: extrinsic epigenetic age acceleration; EAASkinBlood: epigenetic age acceleration with skin and blood clock; GAA: GrimAge acceleration; PAA: PhenoAge acceleration.

Supplementary Table 8. Association between long-term average alcohol consumption and EAAs with white blood cell compositions in young group.

		Without white blood cell compositions		Adjust for white blood cell compositions	
		β	*p*	β	*p*
Beer	IEAA	−0.42	0.30	−0.35	0.39
	EEAA	−0.06	0.90	0.36	0.36
	EAASkinBlood	−0.04	0.88	0.03	0.91
	GAA	0.09	0.76	0.22	0.45
	PAA	−0.46	0.39	−0.25	0.62
Wine	IEAA	−1.97	0.006	−2.07	0.004
	EEAA	0.82	0.33	0.73	0.30
	EAASkinBlood	−0.79	0.09	−0.79	0.09
	GAA	0.79	0.15	0.78	0.13
	PAA	−0.92	0.32	−0.89	0.29
Liquor	IEAA	−0.27	0.62	−0.25	0.64
	EEAA	0.35	0.58	0.71	0.18
	EAASkinBlood	0.03	0.93	0.11	0.76
	GAA	0.13	0.74	0.15	0.68
	PAA	−0.40	0.56	−0.23	0.72
Total alcohol	IEAA	−0.51	0.06	−0.52	0.05
	EEAA	0.06	0.84	0.31	0.22
	EAASkinBlood	−0.08	0.65	−0.04	0.83
	GAA	0.09	0.67	0.16	0.41
	PAA	−0.42	0.23	−0.27	0.39

All associations were adjusted for sex, physical activity score, education level, BMI, smoke pack-year, chronological age, and lab. Models with white blood cells additionally adjusted for CD8+ T, CD4+ T, natural killer cells, B cells, monocytes, and granulocytes. All models contained same set of samples (n = 690). The β coefficients represented the increase in EAA with one additional drink of long-term average alcohol consumption in the young group. Abbreviations: IEAA: intrinsic epigenetic age acceleration; EEAA: extrinsic epigenetic age acceleration; EAASkinBlood: epigenetic age acceleration with skin and blood clock; GAA: GrimAge acceleration; PAA: PhenoAge acceleration.

Supplementary Table 9. Association between long-term average alcohol consumption and EAAs with white blood cell compositions in middle-aged group.

		Without white blood cell compositions		Adjust for white blood cell compositions	
		β	*p*	β	*p*
Beer	IEAA	−0.14	0.40	−0.14	0.39
	EEAA	0.02	0.92	0.18	0.26
	EAASkinBlood	−0.01	0.89	−0.02	0.84
	GAA	0.46	4.3E-04	0.46	2.6E-04
	PAA	0.49	0.02	0.54	0.006
Wine	IEAA	−0.11	0.68	−0.12	0.65
	EEAA	0.09	0.78	0.75	0.005
	EAASkinBlood	0.28	0.11	0.30	0.08
	GAA	0.54	0.01	0.73	5.1E-04
	PAA	0.93	0.007	1.35	3.8E-05

Liquor	IEAA	0.22	0.46	0.21	0.47
	EEAA	−0.03	0.93	0.23	0.43
	EAASkinBlood	−0.09	0.62	−0.10	0.61
	GAA	0.82	4.2E-04	0.89	7.4E-05
	PAA	1.47	6.2E-05	1.57	6.9E-06
Total alcohol	IEAA	−0.04	0.75	−0.04	0.76
	EEAA	−0.07	0.58	0.19	0.10
	EAASkinBlood	0.01	0.92	0.02	0.78
	GAA	0.45	2.8E-06	0.51	2.9E-08
	PAA	0.72	1.6E-06	0.85	2.7E-09

All associations were adjusted for sex, physical activity score, education level, BMI, smoke pack-year, chronological age, and lab. Models with white blood cells additionally adjusted for CD8+ T, CD4+ T, natural killer cells, B cells, monocytes, and granulocytes. All models contained same set of samples (n = 1852). The β coefficients represented the increase in EAA with one additional drink of long-term average alcohol consumption in the middle-aged group. Abbreviations: IEAA: intrinsic epigenetic age acceleration; EEAA: extrinsic epigenetic age acceleration; EAASkinBlood: epigenetic age acceleration with skin and blood clock; GAA: GrimAge acceleration; PAA: PhenoAge acceleration.

Supplementary Table 10. Association between long-term average alcohol consumption and EAAs with white blood cell compositions in older group.

		Without white blood cell compositions		Adjust for white blood cell compositions	
		**β**	***p***	**β**	***p***
Beer	IEAA	−0.19	0.36	−0.14	0.50
	EEAA	−0.14	0.54	0.05	0.79
	EAASkinBlood	−0.06	0.63	−0.03	0.81
	GAA	0.27	0.09	0.31	0.05
	PAA	0.25	0.35	0.37	0.14
Wine	IEAA	−0.27	0.43	−0.27	0.44
	EEAA	−0.48	0.20	−0.30	0.38
	EAASkinBlood	−0.28	0.18	−0.30	0.15
	GAA	−0.01	0.98	0.07	0.79
	PAA	0.99	0.02	1.13	0.005
Liquor	IEAA	0.31	0.28	0.35	0.22
	EEAA	−0.08	0.79	0.13	0.65
	EAASkinBlood	0.05	0.79	0.06	0.73
	GAA	0.91	2.4E-05	0.93	5.7E-06
	PAA	0.81	0.02	0.95	0.004
Total alcohol	IEAA	0.01	0.96	0.04	0.79
	EEAA	−0.10	0.50	0.09	0.54
	EAASkinBlood	−0.03	0.75	−0.004	0.96
	GAA	0.37	8.1E-04	0.41	1.3E-04
	PAA	0.62	5.5E-04	0.75	1.4E-05

All associations were adjusted for sex, physical activity score, education level, BMI, smoke pack-year, chronological age, and lab. Models with white blood cells additionally adjusted for CD8+ T, CD4+ T, natural killer cells, B cells, monocytes, and granulocytes. All models contained same set of samples (n = 1255). The β coefficients represented the increase in EAA with one additional drink of long-term average alcohol consumption in the older group. Abbreviations: IEAA: intrinsic epigenetic age acceleration; EEAA: extrinsic epigenetic age acceleration; EAASkinBlood: epigenetic age acceleration with skin and blood clock; GAA: GrimAge acceleration; PAA: PhenoAge acceleration.

Supplementary Table 11. Association between long-term average alcohol consumption and EAAs with white blood cell compositions in pooled samples.

		Without white blood cell compositions		Adjust for white blood cell compositions	
		**Estimate**	***p***	**Estimate**	***p***
Beer	IEAA	−0.18	0.14	−0.16	0.19
	EEAA	−0.04	0.75	0.15	0.20
	EAASkinBlood	−0.02	0.79	−0.01	0.93
	GAA	0.38	9.2E-05	0.40	1.6E-05
	PAA	0.36	0.02	0.45	0.002
Wine	IEAA	−0.27	0.18	−0.29	0.15
	EEAA	−0.07	0.76	0.36	0.07
	EAASkinBlood	0.00	0.97	0.02	0.88
	GAA	0.32	0.05	0.44	0.003
	PAA	0.88	5.2E-04	1.15	1.6E-06
Liquor	IEAA	0.21	0.27	0.21	0.27
	EEAA	−0.05	0.82	0.16	0.37
	EAASkinBlood	−0.01	0.93	7.3E-04	1.00
	GAA	0.82	2.2E-08	0.81	5.4E-09
	PAA	0.95	4.3E-05	1.03	2.4E-06
Total alcohol	IEAA	−0.06	0.49	−0.06	0.50
	EEAA	−0.07	0.49	0.17	0.05
	EAASkinBlood	−0.004	0.94	0.02	0.75
	GAA	0.40	5.9E-09	0.45	1.0E-11
	PAA	0.61	1.4E-08	0.74	5.7E-13

All associations were adjusted for sex, physical activity score, education level, BMI, smoke pack-year, chronological age, and lab. Models with white blood cells additionally adjusted for CD8+ T, CD4+ T, natural killer cells, B cells, monocytes, and granulocytes. All models contained same set of samples (n = 3797). The β coefficients represented the increase in EAA with one additional drink of long-term average alcohol consumption in the pooled sample. Abbreviations: IEAA: intrinsic epigenetic age acceleration; EEAA: extrinsic epigenetic age acceleration; EAASkinBlood: epigenetic age acceleration with skin and blood clock; GAA: GrimAge acceleration; PAA: PhenoAge acceleration.

Supplementary Table 12. Association between long-term average alcohol consumption and EAAs with long-term covariates.

		Young		Middle-aged		Older		Pooled	
		**β**	***p***	**β**	***p***	**β**	***p***	**β**	***p***
Beer	IEAA	−0.29	0.48	−0.10	0.52	−0.23	0.28	−0.16	0.20
	EEAA	−0.07	0.88	0.04	0.83	−0.14	0.54	−0.03	0.80
	EAASkinBlood	0.03	0.92	0.01	0.92	−0.06	0.62	−5.0E-04	0.99
	GAA	0.06	0.85	0.35	0.009	0.10	0.51	0.25	0.009
	PAA	−0.43	0.42	0.45	0.03	0.14	0.60	0.30	0.05
Wine	IEAA	−1.95	0.007	−0.05	0.87	−0.24	0.49	−0.23	0.26
	EEAA	0.69	0.42	0.10	0.75	−0.43	0.26	−0.08	0.74
	EAASkinBlood	−0.79	0.09	0.30	0.08	−0.22	0.28	0.04	0.77
	GAA	0.15	0.78	0.33	0.14	−0.28	0.23	−0.003	0.99
	PAA	−1.07	0.24	0.90	0.009	0.97	0.02	0.83	9.9E-04

Liquor	IEAA	−0.21	0.70	0.26	0.38	0.21	0.46	0.22	0.23
	EEAA	0.38	0.55	0.03	0.92	−0.09	0.76	−0.06	0.79
	SkinBlood	0.06	0.87	−0.09	0.64	0.03	0.84	−0.008	0.95
	GAA	0.22	0.58	1.05	1.0E-05	0.31	0.12	0.72	3.8E-07
	PAA	−0.34	0.62	1.49	4.2E-05	0.55	0.12	0.87	1.6E-04
Total alcohol	IEAA	−0.44	0.11	0.01	0.93	−0.03	0.84	−0.03	0.73
	EEAA	0.06	0.85	−0.06	0.68	−0.11	0.50	−0.07	0.49
	EAASkinBlood	−0.05	0.80	0.03	0.66	−0.03	0.77	0.02	0.76
	GAA	0.009	0.97	0.38	9.3E-05	0.08	0.44	0.25	1.7E-04
	PAA	−0.40	0.25	0.71	2.3E-06	0.50	0.006	0.57	1.6E-07

All associations were adjusted for sex, education level, chronological age, lab, and long-term average covariates (i.e., BMI, physical activity score, and smoke pack-year). The β coefficients represented the increase in EAA with one additional drink of long-term average alcohol consumption in each age group and pooled samples. Abbreviations: IEAA: intrinsic epigenetic age acceleration; EEAA: extrinsic epigenetic age acceleration; EAASkinBlood: epigenetic age acceleration with skin and blood clock; GAA: GrimAge acceleration; PAA: PhenoAge acceleration.

Supplementary Table 13. Interaction between long-term total alcohol consumptions and sex.

Outcome	Interaction	Young		Middle-aged		Older		Pooled	
		β	*p*	β	*p*	β	*p*	β	*p*
IEAA	Total alcohol consumption × male	0.38	0.58	0.12	0.28	−0.12	0.33	0.009	0.96
EEAA		−0.95	0.67	0.49	0.31	−0.30	0.36	0.07	0.76
EAASkinBlood		0.05	0.38	0.06	0.18	−0.03	0.20	0.05	0.68
GAA		−0.37	0.44	−0.32	0.22	−0.35	0.26	0.08	0.74
PAA		−0.34	0.74	0.46	0.34	−0.54	0.41	−0.24	0.13

All associations were adjusted for sex, physical activity index, education level, BMI, smoke pack-year, chronological age, and lab with female as the reference. The β coefficients represented the increase in EAA with one additional drink of long-term total alcohol consumption in males, compared to increase in females in each age group or the pooled sample. Abbreviations: IEAA: intrinsic epigenetic age acceleration; EEAA: extrinsic epigenetic age acceleration; EAASkinBlood: epigenetic age acceleration with skin and blood clock; GAA: GrimAge acceleration; PAA: PhenoAge acceleration.

Supplementary Table 14. Interaction between long-term total alcohol consumptions and smoking.

	Young		Middle-aged		Older		Pooled	
	β	*p*	β	*p*	β	*p*	β	*p*
IEAA								
Total alcohol consumption×smoker								
Non-smoker				ref				
Current smoker	0.75	0.20	0.49	0.10	−0.51	0.26	0.21	0.35
Former smoker	−0.03	0.98	0.73	0.004	0.44	0.13	0.61	6.85E-04
EEAA								
Total alcohol consumption×smoker								
Non-smoker				ref				
Current smoker	0.64	0.35	−0.01	0.97	−0.77	0.11	−0.24	0.34
Former smoker	0.11	0.94	−0.11	0.69	−0.39	0.21	−0.23	0.26

EAASkinBlood								
Total alcohol consumption×smoker								
Non-smoker					ref			
Current smoker	0.82	0.03	0.43	0.02	−0.20	0.45	0.24	0.09
Former smoker	−0.16	0.85	0.41	0.01	0.03	0.85	0.22	0.05
GAA								
Total alcohol consumption×smoker								
Non-smoker					ref			
Current smoker	−0.98	0.02	0.15	0.50	0.02	0.95	0.02	0.88
Former smoker	1.14	0.22	−0.07	0.69	0.16	0.41	0.09	0.47
PAA								
Total alcohol consumption×smoker								
Non-smoker					ref			
Current smoker	−0.28	0.71	0.28	0.45	−0.34	0.53	0.04	0.89
Former smoker	2.01	0.22	0.05	0.87	0.60	0.09	0.43	0.06

All associations were adjusted for sex, physical activity index, education level, BMI, smoking status, chronological age, and lab with non-drinker as the reference. The β coefficients represented the increase in EAA with one additional drink of long-term total alcohol consumption in former drinkers or current drinkers, compared to increase in non-drinkers in each age group or the pooled sample. Abbreviations: IEAA: intrinsic epigenetic age acceleration; EEAA: extrinsic epigenetic age acceleration; EAASkinBlood: epigenetic age acceleration with skin and blood clock; GAA, GrimAge acceleration; PAA, PhenoAge acceleration.

Supplementary Table 15. Effects of alcohol consumption and EAAs on hypertension.

		OR	*p*	LCI	UCI
Young	Long-term alcohol consumption				
	Beer	1.23	0.40	0.76	2.00
	Wine	0.48	0.17	0.17	1.37
	Liquor	0.75	0.54	0.30	1.86
	Total alcohol	0.96	0.82	0.68	1.35
	EAA				
	GAA	1.01	0.76	0.95	1.08
	PAA	1.01	0.71	0.97	1.05
Middle-aged	Long-term alcohol consumption				
	Beer	1.19	0.09	0.97	1.46
	Wine	1.40	0.04	1.02	1.91
	Liquor	1.74	0.01	1.13	2.70
	Total alcohol	1.27	0.002	1.09	1.48
	EAA				
	GAA	1.09	8.00E-07	1.05	1.12
	PAA	1.05	1.66E-06	1.03	1.08
Older	Long-term alcohol consumption				
	Beer	1.19	0.22	0.90	1.59
	Wine	1.26	0.25	0.85	1.87
	Liquor	1.06	0.72	0.77	1.48
	Total alcohol	1.15	0.13	0.96	1.37
	EAA				
	GAA	1.04	0.10	0.99	1.09
	PAA	1.03	0.05	1.00	1.06

Pooled	Long-term alcohol consumption				
	Beer	1.19	0.03	1.01	1.39
	Wine	1.28	0.04	1.01	1.61
	Liquor	1.21	0.11	0.96	1.53
	Total alcohol	1.19	0.002	1.07	1.32
	EAA				
	GAA	1.06	2.51E-06	1.03	1.08
	PAA	1.04	2.77E-06	1.02	1.05

All associations were adjusted for sex, physical activity index, education level, BMI, smoke pack-year, chronological age, and lab. Abbreviations: LCI: low confidence interval; UCI: upper confidence interval. Abbreviations: GAA: GrimAge acceleration; PAA: PhenoAge acceleration.

Supplementary Table 16. Mediation analysis of EAAs on the association of long-term alcohol consumption and hypertension.

			Indirect association		
			**β**	***p***	**Mediated percentage (%)**
Young	Beer	GAA	5.15E-04	0.712	1.32
		PAA	−6.70E-04	0.67	−1.71
	Wine	GAA	0.002	0.64	−1.37
		PAA	−0.001	0.68	1.21
	Liquor	GAA	−7.80E-04	0.70	1.75
		PAA	−7.20E-04	0.70	1.63
	Total alcohol	GAA	2.41E-04	0.75	−2.06
		PAA	−6.90E-04	0.65	5.88
Middle-aged	Beer	GAA	0.007	0.004	23.78
		PAA	0.006	0.02	17.65
	Wine	GAA	0.005	0.20	7.29
		PAA	0.008	0.03	12.62
	Liquor	GAA	0.01	0.004	17.52
		PAA	0.01	0.002	19.30
	Total alcohol	GAA	0.007	0.001	16.43
		PAA	0.007	0.001	16.35
Older	Beer	GAA	0.001	0.29	5.59
		PAA	8.60E-04	0.41	4.22
	Wine	GAA	−9.80E-04	0.51	−3.41
		PAA	0.003	0.17	10.71
	Liquor	GAA	0.006	0.12	52.04
		PAA	0.004	0.10	40.99
	Total alcohol	GAA	0.002	0.16	9.95
		PAA	0.002	0.12	11.69

Pooled	Beer	GAA	0.003	0.004	13.67
		PAA	0.002	0.03	9.50
	Wine	GAA	0.001	0.57	2.05
		PAA	0.005	0.01	11.16
	Liquor	GAA	0.008	4.00E-04	27.55
		PAA	0.007	7.00E-04	24.17
	Total alcohol	GAA	0.004	7.00E-04	13.30
		PAA	0.004	6.00E-04	13.33

All associations were adjusted for sex, physical activity index, education level, BMI, smoke pack-year, chronological age, and lab. Abbreviations: GAA: GrimAge acceleration; PAA: PhenoAge acceleration.
